# Entropy generation in magneto couple stress bionanofluid flow containing gyrotactic microorganisms towards a stagnation point on a stretching/shrinking sheet

**DOI:** 10.1038/s41598-023-48676-3

**Published:** 2023-12-05

**Authors:** Muhammad Salim Khan, Zahir Shah, Muhammad Roman, Waris Khan, Narcisa Vrinceanu, Mansoor H. Alshehri

**Affiliations:** 1 Department of Mathematical Sciences, University of Lakki Marwat, Lakki Marwat, 28420 Khyber Pakhtunkhwa Pakistan; 2 Department of Mathematics, Hazara University, Mansehra, KPK Pakistan; 3 Department of Industrial Machines and Equipments, Faculty of Engineering, “Lucian Blaga” University of Sibiu, 10 Victoriei Boulevard, Sibiu, Romania; 4 Department of Mathematics, College of Science, King Saud University, P.O. Box 2455, 11451 Riyadh, Saudi Arabia

**Keywords:** Engineering, Materials science, Mathematics and computing, Nanoscience and technology

## Abstract

The study focuses on the behavior of an electrically conducting non-Newtonian fluid with couple stress properties, using water-based bionanofluid. The fluid is analyzed as it flows across a porous stretching/shrinking sheet within its own plane. This Study also explores the Bejan Number and Entropy Generation. To facilitate this investigation, the governing nonlinear partial differential equations undergo a transformation, wherein they are converted into nonlinear ordinary differential equations through a suitable similarity transformation. An ideal strategy has been employed to achieve the desired results from the modeled challenge. The Homotopy Analysis Method is applied to determine the solution of the system of differential equations. The convergence of the applied method and their comparison with the numerical method are described through graphs and tables. The main features of the different profiles are briefly described. Graphs are used to analyze the impact of the Bejan number, concentration, temperature, velocity profile, and entropy production rate. Tables present the characteristics of skin friction, Nusselt, and Sherwood numbers for various limitations. The stretching and ambient fluid velocities should fluctuate linearly as the distance from the stagnation point increases. A rise in the magnetic and porosity parameters is accompanied by an increase in the velocity profile. While the velocity profile falls off as a Couple of fluid parameters are increased. The phenomenon of temperature boost is observed to be positively correlated with the increase in Brownian motion parameter while exhibiting no significant dependence on other parameters such as Brinkman number, Prandtl number Lewis number and Thermophoresis parameter. Entropy generation increases with the Brinkman number while decreasing with the radiation parameter and diffusion parameter as is plainly demonstrated.

## Introduction

Bionanofluid, a cutting-edge composite material, is defined by its unique composition as a fluid that incorporates meticulously designed artificial nanoparticles sourced from biological origins, and integrated into a fluid medium^1^. These nanoparticles are precision-crafted at the nanoscale and can originate from various biological materials, including proteins, enzymes, DNA, viruses, and other biological molecules^2–4^. Bionanofluids have sparked considerable interest due to their versatile applications in diverse fields such as healthcare, electronics, energy, and environmental science. The fusion of nanoparticles with a fluid matrix leads to the enhancement of multiple fluid properties, encompassing thermal conductivity, electrical conductivity, optical characteristics, and mechanical attributes. This symbiotic integration opens avenues for the development of novel materials with heightened performance and tailored functionalities. In a theoretical exploration, it becomes evident that bionanofluids offer a vast spectrum of applications, ranging from precise drug delivery systems, biosensors, and tissue engineering, to advanced medical imaging techniques. In the realm of drug administration, nanoparticles play a pivotal role by facilitating targeted drug transport to specific locations within the body, enhancing imaging contrast, and providing structural support for tissue regeneration^4,5^. In the electronics sector, bionanofluids contribute to improved heat dissipation, enhanced solar cell efficiency, and the advancement of flexible and transparent displays through superior thermal conductivity and optical properties^1–5^. Furthermore, these fluids find utility in energy conversion and storage systems, such as fuel cells and batteries, where nanoparticles enhance performance, and durability, and serve as catalysts for sustainable chemical reactions^1–5^. The empirical review delves into a range of research endeavors that employ entropy optimization and various methodologies in the field of fluid dynamics. For instance, Rashid et al. ^6^ delve into the application of entropy in the context of ferromagnetic fluid flow, encompassing nonlinear radiative effects and error conditions. Qayyum et al.^7^ explore the phenomenon of entropy groups in dissipative fluids confined between rotating discs. Meanwhile, Zaib et al.^8^ conducted a numerical analysis to scrutinize the entropic behavior of mixed magnetohydrodynamic (MHD) convective currents emerging from non-isothermal flat plates. Farooq et al.^9^ incorporate entropy optimization with slip effects to probe fluid rotation, and Shaw et al.^10^ synergize entropy optimization with Fourier analysis in their research. These studies collectively make invaluable contributions to the existing body of knowledge by applying entropy and optimization techniques to gain deeper insights into fluid behavior across diverse scenarios. Such research has the potential to enhance our understanding of bionanofluid dynamics and its practical implications. Magnetohydrodynamics (MHD) is the scientific study of the interaction of magnetic fields and fluids that conduct electricity. Flow meters, MHG power generation, pumps, and other industrial uses are some of the more notable examples. Over a stretching sheet, Babu and Sandeep^11^ studied the motion of a 3D MHD nanofluid. In addition, Ghadikolaei et al.^12,13^, and Hasio^14^, looked into MHD and other hybrid nanofluids across the stretched sheet. Several scientists have also conducted research in this area^15–17^. The influence of magnetic fields on entropy generation has recently gained prominence within the realm of thermodynamics. In addition to conventional factors like temperature and pressure, magnetic fields have been identified as significant contributors to alterations in a system's entropy. Naveed^18–20^ delves deeper into the subject, building upon extensive research that has explored this phenomenon across various scientific disciplines. The imperative of accounting for magnetic field effects in the context of entropy generation becomes evident. This understanding is vital for achieving precise models and predictions of complex systems, particularly those involving magnetic materials or processes. As we continue to navigate the intricate realms of thermodynamics and materials science, recognizing the pivotal role of magnetic fields in entropy generation becomes an indispensable aspect of advancing our comprehension of physical and chemical processes. Stagnation points are of paramount significance in the realm of fluid dynamics due to their association with local fluid velocity reaching zero. This phenomenon has found vital applications in both industrial and scientific domains. Recent research has harnessed stagnation points to manipulate the movement of molecules and particles within fluid flows, leading to significant advancements in chemistry, biology, and medicine^21–23^. The foundational concept of two-dimensional stagnation point flow against a solid wall was introduced by Hiemenz^24, 25^. Subsequently, Ariel^26^ proposed an approximation method to address stagnation-related issues, while Mahapatra and Gupta^27,28^ and Wang^29,30^ independently formulated the concept of two-dimensional stagnation point flow over plates with either expansion or contraction capabilities. The coexistence of stagnation point flow and surface deformation results in intricate flow patterns, influencing velocity, heat, and mass transfer rates within the boundary layer flow, thus creating a complex flow structure. Shrinkage in fluid dynamics holds considerable importance in technology and engineering. The concept of couple stresses, originally introduced by Stokes^31^, was developed to address the unique rheological characteristics of complex non-Newtonian fluids, which manifest body stresses and couples that fall outside the purview of traditional continuum mechanics. The utilization of such fluids in diverse sectors like engineering, biology, and chemistry has garnered significant attention^32–34^. Furthermore, thermal radiation plays a pivotal role in various industrial applications, including industrial electricity generation, solar power technology, glass manufacturing, and furnace design. Researchers like Dogonch et al.^35^ and Reddy et al.^36^ have investigated the impact of heat radiation on nanofluids, particularly Fe3O4-H2O nanofluids and Jeffrey nanofluids. Numerous studies have explored the effects of thermal radiation on Casson fluids flowing over large surfaces. Additionally, researchers such as Ghadikolaei et al.^37^ and Oyelakin et al.^38^, have delved into the optimization of entropy generation in thermodynamic systems, focusing on the use of the tangent-hyperbolic nanomaterial model ^39^, to enhance thermo-transmissions. The impetus behind studying stagnation points arises from the observation that in their vicinity, there is a significant increase in heat and mass transfer rates. Consequently, scholars are highly motivated to comprehend the behavior of bionanofluid flows as they approach a stagnation point on a diminishing surface. The current context involves the examination of numerous parameters, including velocity and thermal slippage in close proximity to the shrinking surface^40^.

The flow of nanofluids on stretching/shrinking surfaces has gained significant prominence in fluid dynamics due to its pertinence in diverse industrial and scientific applications. A crucial aspect of this flow is the inclusion of Couple stress effects, which play a pivotal role in determining the behavior and characteristics of the nanofluid flow^41,42^. Couple stress fluids, also known as micropolar fluids, exhibit additional internal rotational characteristics compared to conventional Newtonian fluids. These additional internal rotational characteristics arise from the microscale interactions between particles or molecules within the fluid. Recently, there has been a burgeoning interest in studying the flow of a Couple of stress nanofluids on stretching/shrinking surfaces. This interest stems from the broad spectrum of applications where a Couple stress fluids can be found, such as in squeezing and lubrication, bio-fluid mechanics, MHD flows, and the synthesis and plasticity of chemical compounds. Researchers have conducted numerous studies to investigate the effects of couple stress on nanofluid flow over stretching/shrinking surfaces^43^. In a study conducted by Awais et al.^44^, they investigated the characteristics of nanofluids with couple stresses under the influence of Newtonian heating in a vertical setup. Furthermore, Hayat et al.^45^ contributed to the field by presenting an extensive analysis of Magnetohydrodynamics, incorporating the concept of couple stress nanofluids. Hayat et al.^45^ conducted a study in which they analyzed the flow of two-dimensional incompressible micropolar nanofluids over a linearly stretching or shrinking sheet, taking into account the effects of velocity, thermal, and concentration slip. The significance of incorporating Couple stress effects into nanofluid flow on stretching or shrinking surfaces becomes apparent for several reasons. First, it improves the accuracy of predictions in heat transfer applications. This is because a Couple stress fluids exhibit enhanced heat transfer properties compared to Newtonian fluids. Second, the study of Couple stress nanofluid flow can provide valuable insights into the behavior and performance of systems involving stretching/shrinking surfaces. For instance, in the cooling of metallic plates in a bath, the presence of Couple stress effects can influence the rate of heat transfer between the plate and the fluid. Overall, the study of Couple stress nanofluid flow on stretching/shrinking surfaces is a significant area of research with a wide range of potential applications.

The key objective of this study is to investigate the generation of entropy and the impact of modifying the fluid on the stagnation point. The energy relationship has been conceptualized and represented utilizing a diverse range of variables. The equations pertaining to the flow problem are elucidated and subsequently modified through the appropriate comparability transformation. Utilizing similarity transformations leads to the transformation of the governing partial differential equations into a system of ordinary differential equations. To ascertain the convergence of the Homotopy Analysis Method (HAM), numerical analysis has been applied. It is analyzed and highlights how important certain characteristics are in various profiles. A graphic analysis of the impacts of the Bejan number, concentration, temperature, velocity profile, and entropy production rate is presented. The features of Sherwood number, Nusselt number, and skin friction for different limitations are displayed in tables. This novel and realistic mathematical viewpoint on stagnation point flow, along with numerical results, should close a gap in the literature and serve as an all-inclusive fundamental technique for upcoming laboratory work and the production of biomedical devices (biosensors). A physical event that includes both a stagnation point and a decreasing surface flow is one that is legitimate and dependable. Our paper is organized in the following ways.

## Mathematical formulation

**Figure 1:**
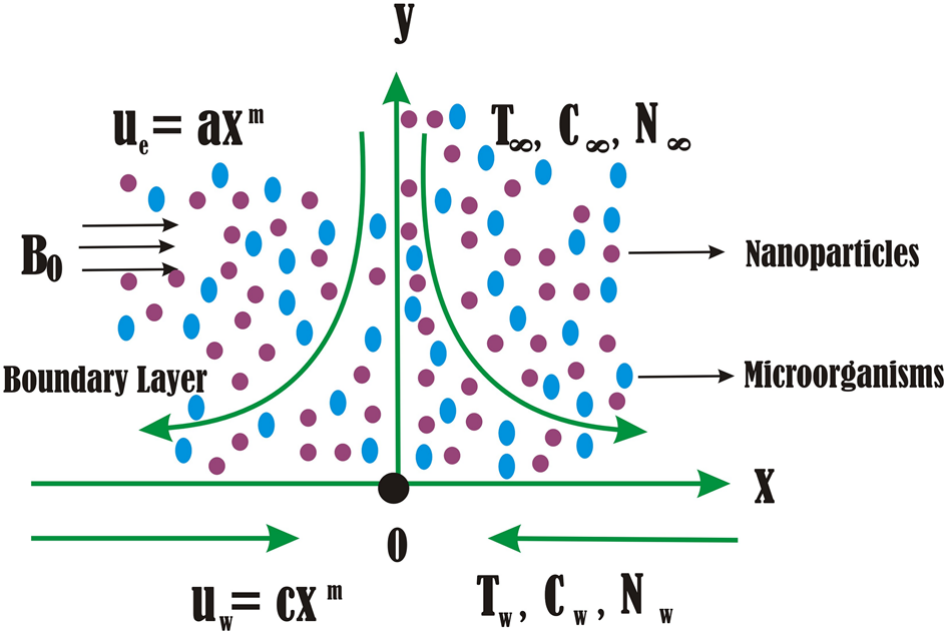
Physical sketch of the flow.

In this study we considered the incompressible steady couple stress electrically conducting bionanofluid over a permeable stretching/shrinking surface in the existence of microorganisms. The geometry of the problem (shown in Fig. 1) is designed that the coordinate system (*x*, *y*) chosen such as *y* > 0 region, which is induced. The surface is characterised by a stationary stagnation point located at *x* = 0 *y* = 0 where the componenst of velocity *u* is parallel and *v* is normal stretching/shrinking surface. The shrinking surface velocity immersed in the bionanofluid flow is represented and defined as $u_{w} (x) = cx^{m}$ and the free flow velocity is $u_{e\left( x \right)} = ax^{m}$, where *m* = 1 (represent linear case) & a > 0, both velocities are varying. In the case of a declining surface with a constant negative value of c, the variables $u_{w\left( x \right)} , u_{e\left( x \right)}$, and $v_{w\left( x \right)}$ denote the velocities associated with stretching or shrinking, the ambient fluid, and the mass flux, respectively. Also we consider $v_{w\left( x \right)}$ as a continuous mass flux velocity with the following conditions: suction = $v_{w\left( x \right)} < 0$ injection = $v_{w\left( x \right)} > 0$. It is assumed that the variable T denotes the uniform temperature, C denotes the uniform volume fraction of nanofluid, N denotes the uniform concentration of microbes, and *B*_0_ denotes Magnetohydrodynamics effect.

### The governing equations with conditions

The governing equations of the proposed model after applying assumptions are designed in the following ways^40^:

1 $$\frac{\partial u}{{\partial x}} + \frac{\partial v}{{\partial y}} = 0,$$

Equation of momentum

2 $$u\frac{\partial u}{{\partial x}} + v\frac{\partial u}{{\partial y}} = u_{e} \frac{{du_{e} }}{dx} + \nu \frac{{\partial^{2} u}}{{\partial y^{2} }} - \frac{\nu ^{\prime}}{\rho }\frac{{\partial^{4} u}}{{\partial y^{4} }} - \frac{\nu }{k}u - \frac{{\sigma_{1} B_{0}^{2} }}{\rho }u,$$

Equation of thermal energy

3 $$u\frac{\partial T}{{\partial x}} + v\frac{\partial T}{{\partial y}} = \begin{array}{*{20}c} {\alpha \left( {1 + \frac{{16\sigma^{*} T_{\infty }^{3} }}{{3k^{*} k}}} \right)\frac{{\partial^{2} T}}{{\partial y^{2} }} + \tau \left[ {D_{B} \frac{\partial C}{{\partial Y}}\frac{\partial T}{{\partial y}} + \left( {\frac{{D_{T} }}{{T_{\infty } }}} \right)\left( {\frac{\partial T}{{\partial y}}} \right)^{2} } \right]} \\ { + \frac{\nu }{\rho cp}\left( {\frac{\partial u}{{\partial y}}} \right)^{2} + \frac{\nu ^{\prime}}{{\rho cp}}\left( {\frac{{\partial^{2} u}}{{\partial y^{2} }}} \right)^{2} + \frac{{\sigma_{1} B_{0}^{2} }}{\rho cp}u^{2} } \\ \end{array} ,$$

Equation of nanoparticle volume fraction

4 $$u\frac{\partial C}{{\partial x}} + v\frac{\partial C}{{\partial y}} = D_{B} \frac{{\partial^{2} C}}{{\partial y^{2} }} + \left( {\frac{{D_{T} }}{{T_{\infty } }}} \right)\frac{{\partial^{2} T}}{{\partial y^{2} }},$$

Equation of microorganism density

5 $$u\frac{\partial N}{{\partial x}} + v\frac{\partial N}{{\partial y}} + \frac{\partial }{\partial y}\left( {N\tilde{v}} \right) = D_{n} \frac{{\partial^{2} N}}{{\partial y^{2} }}.$$

Here kinematic viscosity is *ν*, nanoparticle thermal diffusivity is *α*, the proportion of nanoparticle's heat capacity is *τ*, while Brownian diffusion coefficient are *D*_*B*_, *D*_*n*_ and *D*_*τ*_.

The thermophoretic diffusion coefficient and microorganism diffusivity will be discussed in this context. $\tilde{v} = \frac{{bW_{c} }}{{(C_{w} - C_{\infty } )}}(\partial C/\partial y)$. The relationship between the chemotaxis constant (b) and the maximal swimming speed (Wc) of a microbe is elucidated.

The boundary conditions are^40^:

6 $$\left. {\begin{array}{*{20}l} {u = N_{1} \frac{\partial u}{{\partial y}} + u_{w} ,\quad v = v_{w} \left( x \right),\quad T = D_{1} \frac{\partial T}{{\partial y}} + T_{w} ,} \hfill \\ {N = N_{w} ,\quad C = C_{w} \quad at,\quad y \to 0,} \hfill \\ {T = T_{\infty } ,\quad u = u_{e} \left( x \right),\quad N = N_{\infty } ,\quad C = C_{\infty } , \quad as\quad y \to \infty ,} \hfill \\ \end{array} } \right\}$$

where *v*_*w*_(*x*) < 0 shows suction, *v*_*w*_(*x*) > 0 shows injection, *c* < 0 resembles to shrinking surface, while $u_{w} \left( x \right) = cx^{m}$, and $v_{w} \left( x \right) = - \frac{m + 1}{2}\left( {\frac{{u_{e} \left( x \right)\nu }}{x}} \right)^{\frac{1}{2}} S$. Here, *a*, *c* and *m* are numbers whrer *m* = 1, & *a* > 0 (linear case).

### Similarity variables and transformations

The following similarity variables are introduced^40^:

7 $$\left. {\begin{array}{*{20}l} {\eta = \left( {\frac{{u_{e} \left( x \right)}}{vx}} \right)y,\quad \psi = \left( {u_{e} \left( x \right)vx} \right)^{1/2} f\left( \eta \right),} \hfill \\ {\theta \left( \eta \right) = \left( {\frac{{T - T_{\infty } }}{\Delta T}} \right), } \hfill \\ {\phi \left( \eta \right) = \left( {\frac{{C - C_{\infty } }}{\Delta C}} \right),\quad \chi \left( \eta \right) = \left( {\frac{{N - N_{\infty } }}{\Delta N}} \right)} \hfill \\ \end{array} } \right\}$$

where steam function *ψ* defined as: $u = \frac{\partial \psi }{{\partial y}},v = - \frac{\partial \psi }{{\partial x}} ,\Delta C = C_{w} - C_{\infty } ,\Delta N = N_{w} - N_{\infty }$, and $\Delta T = T_{w} - T_{\infty }$, The velocities are $u = u_{e} \left( x \right)f^{\prime } \left( \eta \right)$ and $v = - \frac{m + 1}{2}\left( {\frac{{u_{e} \left( x \right)\nu }}{x}} \right)$.

The aforementioned similarity variable is utilized to derive the subsequent ordinary differential equations along with the boundary conditions from the set of governing equations:

8 $$f^{\prime \prime \prime } - \frac{1}{{\lambda_{1} }}f^{\prime \prime \prime \prime \prime } - Mf^{\prime } - \beta_{0} f^{\prime } f^{\prime 2} + f^{\prime \prime } f + 1 = 0$$

9 $$\left( {1 + Rd} \right)\theta^{\prime \prime } + Pr\left[ {f\theta^{\prime} + Nb\theta^{\prime } \phi^{\prime } + Nt\theta^{{^{{\prime}{2}} }} } \right] + Brf^{{^{\prime \prime 2} }} + \frac{Br}{{\lambda_{1} }}f^{{^{\prime \prime \prime 2} }} + MBrf^{{^{\prime 2} }} = 0$$

10 $$\phi^{\prime \prime } + \frac{Nt}{{Nb}}\theta^{\prime } + Lef\phi^{\prime } = 0$$

11 $$\chi^{\prime \prime } + Scf\chi^{\prime } - Pe\left[ {\left( {\sigma + \chi } \right)\phi^{\prime \prime } + \phi^{\prime } \chi^{\prime } } \right] = 0$$

12 $$f^{\prime } \left( 0 \right) = \gamma f^{\prime \prime } \left( 0 \right) + \lambda ,\quad f\left( 0 \right) = S,\quad \phi \left( 0 \right) = 1,\quad \theta \left( 0 \right) = 1 + \beta \theta^{\prime } \left( 0 \right),$$

13 $$f^{\prime } \left( \infty \right) = 1,\quad \chi \left( 0 \right) = 1,\quad \chi \left( \infty \right) = 0, \quad \phi \left( \infty \right) = 0,\quad \theta \left( \infty \right) = 0.$$

where *λ* < 0 and used for shrinking parameters. The slip factors are epitomized as $\beta = D_{1} \left( {\frac{{u_{e} \left( x \right)}}{vx}} \right)^{1/2}$ and $\lambda = N_{1} \left( {\frac{{u_{e} \left( x \right)}}{vx}} \right)^{1/2}$. The model is subject to various parameters, including Prandtl number (Pr), Lewis number (Le), Péclet number for bioconvection (Pe), and Schmidt number (Sc). λ_1_ which represents the fluid couple, thermophoresis parameter (Nt), dimensionless constant (r), and Brownian motion parameter (Nb). The aforementioned parameters are of paramount importance in governing the system.

14 $$\left. {\begin{array}{*{20}l} {Pr = \frac{\nu }{\alpha },\quad Ec = \frac{{u_{e}^{2} }}{{c_{p} \left( {T_{w} - T_{\infty } } \right)}},\quad B_{r} = P_{r} \cdot Ec,\quad Le = \frac{\nu }{{D_{B} }},} \hfill \\ {Pe = \frac{{bW_{c} }}{{D_{n} }},\quad Sc = \frac{\nu }{{D_{n} }},\quad Nb = \frac{{\tau D_{B} \left( {C_{w} - C_{\infty } } \right)}}{\nu },\quad M = - \frac{{\sigma B_{0}^{2} }}{\rho a}} \hfill \\ {NT = \frac{{\tau D_{\tau } \left( {C_{w} - C_{\infty } } \right)}}{{\nu T_{\infty } }},\quad \sigma = \frac{{N_{\infty } }}{{N_{w} - N_{\infty } }},\quad Rd = \frac{{16\sigma^{*} T_{\infty }^{3} }}{{3k^{*} k}},\quad \lambda_{1} = \frac{{\nu^{\prime } }}{{\nu^{2} }}a^{2} x} \hfill \\ \end{array} } \right\}$$

## Physical quantities

The skin friction coefficient (*C*_*fx*_), which serves to quantify the frictional drag experienced by the fluid flow along the surface. Moreover, the convective heat transfer at a specific location is quantified using the local Nusselt number (*Nu*_*x*_), which establishes a relationship between the heat transfer coefficient and thermal conductivity. Similarly, the local Sherwood number (*Sh*_*x*_) characterizes convective mass transfer at a particular location, connecting the mass transfer coefficient to the species' diffusivity. Finally, the concentration of actively moving microorganisms near the surface is denoted by the local density of motile microorganisms (*Nn*_*x*_).

15 $$\left. \begin{gathered} C_{fx} = \frac{{\tau_{w} }}{{\rho u_{e}^{2} }},\quad Nu_{x} = \frac{{xq_{w} }}{{k\left( {T_{w} - T_{\infty } } \right)}}, \hfill \\ Sh_{x} = \frac{{xq_{m} }}{{D_{B} \left( {C_{w} - C_{\infty } } \right)}},\quad Nn_{x} = \frac{{xq_{n} }}{{D_{n} \left( {N_{w} - N_{\infty } } \right)}} \hfill \\ \end{gathered} \right\}$$

where $\tau_{\omega } = \nu \left( {\frac{\partial u}{{\partial y}}} \right)_{y = 0} - \nu^{\prime } \left( {\frac{{\partial^{3} u}}{{\partial y^{3} }}} \right)_{y = 0} ,q_{w} = - k\left( {\frac{\partial T}{{\partial y}}} \right)_{y = 0} ,q_{m} = - D_{B} (\partial C/\partial y)_{y = 0} ,q_{n} = - D_{n} (\partial N/\partial y)_{y = 0}$. These terms are employed to describe surface shear stress, wall heat flux, wall mass flux, and the flux of motile microorganisms at the wall. By using Eqs. (7), (13) and (14) we obtained:

16 $$\left. {\begin{array}{*{20}l} {\sqrt {Re_{x} } C_{fx} = f^{\prime\prime}\left( 0 \right) - \lambda_{1} f^{^{\prime\prime\prime\prime}} \left( 0 \right)} \hfill \\ {} \hfill \\ { - \theta^{\prime}\left( 0 \right) = (Re_{x} )^{ - 1/2} Nu_{x} } \hfill \\ {} \hfill \\ { - \phi^{\prime}\left( 0 \right) = (Re_{x} )^{ - 1/2} Sh_{x} } \hfill \\ {} \hfill \\ { - \chi^{\prime}\left( 0 \right) = (Re_{x} )^{ - 1/2} Nn_{x} } \hfill \\ {} \hfill \\ {} \hfill \\ \end{array} } \right\}$$

## Entropy generation and Bejan number

To grasp the irreversibility of thermal energy within a system, it is essential to take into account the generation of entropy in all systems. The current model's main objective is to enhance outcomes by lowering the creation of entropy through the modification of numerous physical parameters. The mathematical method below can be used to get the entropy production ratio per unit volume for the current model^18,46^.

17 $$S_{g} = \left. {\begin{array}{*{20}c} {\frac{k}{{T_{\infty }^{2} }}\left( {1 + \frac{{16\sigma^{*} T_{\infty }^{3} }}{3k*k}} \right)\left( {\frac{\partial T}{{\partial y}}} \right)^{2} + \frac{{RD_{B} }}{{C_{\infty } }}\left( {\frac{\partial C}{{\partial y}}} \right)^{2} } \\ { + \frac{{RD_{B} }}{{T_{\infty } }}\left( {\frac{\partial C}{{\partial y}}\frac{\partial T}{{\partial y}}} \right) + \frac{v}{{T_{\infty } }}\left( {\frac{\partial u}{{\partial y}}} \right)^{2} + \frac{{v^{{\prime }} }}{{T_{\infty } }}\left( {\frac{{\partial^{2} u}}{{\partial y^{2} }}} \right)^{2} + \frac{{\sigma_{1} B_{0}^{2} }}{{T_{\infty } }}u^{2} } \\ \end{array} } \right\}$$

By using the similarity transformation Eq. (7) we get the following result.

18 $$N_{G} = \left( {\alpha_{1} - 1} \right)\left( {1 + Rd} \right)\theta^{{^{{{\prime }2}} }} + L\frac{{(\alpha_{2} - 1)^{2} }}{{\alpha_{1} - 1}}\phi^{{^{{{\prime }2}} }} + L\left( {\alpha_{2} - 1} \right)\phi^{{\prime }} \theta^{{\prime }} + \frac{MBr}{{\alpha_{1} }}f^{{^{{{\prime }2}} }} + Brf^{{{\prime \prime }}} + \lambda_{1} Brf^{{^{{{\prime \prime \prime }2}} }}$$

The Bejan number, denoted as *Be*, is a non-dimensional quantity that finds its application in the fields of fluid dynamics and thermodynamics. The parameter denotes the proportion of irreversibility in heat transfer to that in fluid flow within a given system. The nomenclature "Bejan number" is attributed to Adrian Bejan, a distinguished physicist and scholar who has made significant contributions to the domains of thermodynamics and heat transfer. The aforementioned statement offers valuable insights regarding the comparative importance of thermal and flow irreversibilities within a specific system.

19 $$Be = \frac{{\left( {\alpha_{1} - 1} \right)\left( {1 + Rd} \right)\theta^{{^{\prime 2} }} }}{{N_{G} }}$$

## Methodology

Applying the Homotopy Analysis Method (HAM) and the given boundary conditions (12, 13), we are able to determine the solutions of Eqs. (8–11). The inclusion of auxiliary parameters helps set and optimize the convergence. For the model Eqs. (8–11), we find the primary guesses as

20 $$f_{0} \left( \eta \right) = s + \eta - \eta e^{ - \eta } ,\quad \theta_{0} \left( \eta \right) = \frac{1}{1 + \beta }e^{ - \eta } ,\quad \phi_{0} \left( \eta \right) = e^{ - \eta } ,\quad \chi_{0} \left( \eta \right) = e^{ - \eta }$$

where the linear operators are defined as:

21 $$L_{f} \left( f \right) = \frac{{\partial^{3} f}}{{\partial \eta^{3} }} - \frac{\partial f}{{\partial \eta }},\quad {\text{L}}_{\theta } \left( \theta \right) = \frac{{\partial^{2} \theta }}{{\partial \eta^{2} }} - \theta ,\quad L_{\phi } \left( \phi \right) = \frac{{\partial^{2} \phi }}{{\partial \eta^{2} }} - \phi ,\quad L_{\chi } \left( \chi \right) = \frac{{\partial^{2} \chi }}{{\partial \eta^{2} }} - \chi$$

Which have the following properties:

22 $$\begin{array}{*{20}l} &{ L_{f} \left( {{\rm K}_{1} + {\rm K}_{2} e^{ - \eta } + {\rm K}_{3} e^{\eta } } \right) = 0,\quad {\text{L}}_{\theta } \left( {{\rm K}_{4} e^{ - \eta } + {\rm K}_{5} e^{\eta } } \right) = 0,} \hfill \\ & { L_{\phi } \left( {{\rm K}_{6} e^{ - \eta } + {\rm K}_{7} e^{\eta } } \right) = 0,\quad L_{\chi } \left( {{\rm K}_{8} e^{ - \eta } + {\rm K}_{9} e^{\eta } } \right) = 0} \hfill \\ \end{array}$$

The solution in its entirety comprises undetermined constants, represented as $c_{i} \left( {i = 1 - 9} \right)$, which are ascertained in accordance with the particular constraints of the given problem. The non-linear operators ${\text{N}}_{f} ,N_{\theta } , N_{\phi } ,N_{\chi }$ that ensue are formulated as:

23 $$\begin{aligned} N_{f} \left[ {f\left( {\eta ;p} \right)} \right] & = \frac{{\partial^{3} f\left( {\eta ;p} \right)}}{{\partial \eta^{3} }} - \frac{1}{{\lambda_{1} }}\frac{{\partial^{5} f\left( {\eta ;p} \right)}}{{\partial \eta^{5} }} - M\frac{{\partial f\left( {\eta ;p} \right)}}{\partial \eta } \\ & \quad - \beta_{0} \left( {\frac{{\partial f\left( {\eta ;p} \right)}}{\partial \eta }} \right)^{2} + f\left( {\eta ;p} \right)\frac{{\partial^{2} f\left( {\eta ;p} \right)}}{{\partial \eta^{2} }} + 1, \\ \end{aligned}$$

24 $$\begin{array}{*{20}l} &{ N_{\theta } \left[ {f\left( {\eta ;p} \right),\theta \left( {\eta ;p} \right),\theta \left( {\eta ;p} \right)} \right] = \left( {1 + Rd} \right)\frac{{\partial^{2} \theta \left( {\eta ;p} \right)}}{{\partial \eta^{2} }} + Br\left( {\frac{{\partial^{2} f\left( {\eta ;p} \right)}}{{\partial \eta^{2} }}} \right)^{2} + \frac{Br}{{\lambda_{1} }}\left( {\frac{{\partial^{3} f\left( {\eta ;p} \right)}}{{\partial \eta^{3} }}} \right)^{2} } \hfill \\ & { \Pr \left( {f\left( {\eta ;p} \right)\frac{{\partial \theta \left( {\eta ;p} \right)}}{\partial \eta } + Nb\frac{{\partial \theta \left( {\eta ;p} \right)}}{\partial \eta }\frac{{\partial \phi \left( {\eta ;p} \right)}}{\partial \eta } + Nt\left( {\frac{{\partial \theta \left( {\eta ;p} \right)}}{\partial \eta }} \right)^{2} } \right),} \hfill \\ \end{array}$$

25 $$N_{\phi } \left[ {f\left( {\eta ;p} \right),\theta \left( {\eta ;p} \right),\phi \left( {\eta ;p} \right)} \right] = \frac{1}{Sc}\frac{{\partial^{2} \phi \left( {\eta ;p} \right)}}{{\partial \eta^{2} }} + \frac{Nt}{{Nb}}\frac{{\partial^{2} \phi \left( {\eta ;p} \right)}}{{\partial \eta^{2} }} + Lef\left( {\eta ;p} \right)\frac{{\partial \phi \left( {\eta ;p} \right)}}{\partial \eta }$$

26 $$\begin{aligned} N_{\chi } \left[ {f\left( {\eta ;p} \right),\phi \left( {\eta ;p} \right),\chi \left( {\eta ;p} \right)} \right] & = \frac{{\partial^{2} \chi \left( {\eta ;p} \right)}}{{\partial \eta^{2} }} + Lef\left( {\eta ;p} \right)\frac{{\partial \chi \left( {\eta ;p} \right)}}{\partial \eta } + \frac{Nt}{{Nb}}\frac{{\partial^{2} \phi \left( {\eta ;p} \right)}}{{\partial \eta^{2} }} \\ & \quad - Pe\left( {\sigma \frac{{\partial^{2} \phi \left( {\eta ;p} \right)}}{{\partial \eta^{2} }} + \chi \left( {\eta ;p} \right)\frac{{\partial^{2} \phi \left( {\eta ;p} \right)}}{{\partial \eta^{2} }} + \frac{{\partial \phi \left( {\eta ;p} \right)}}{\partial \eta }\frac{{\partial \chi \left( {\eta ;p} \right)}}{\partial \eta }} \right) \\ \end{aligned}$$

The *m*th-order problem satisfies the following:

27 $$\begin{aligned} L_{f} \left[ {f_{m} \left( \eta \right) - X_{m} f_{m - 1} \left( \eta \right)} \right] & = \hbar_{f} R_{m}^{f} \left( \eta \right),L_{\theta } \left[ {\theta_{m} \left( \eta \right) - X_{m} \theta_{m - 1} \left( \eta \right)} \right] = \hbar_{\theta } R_{m}^{\theta } \left( \eta \right), \\ L_{\phi } \left[ {\phi_{m} \left( \eta \right) - X_{m} \phi_{m - 1} \left( \eta \right)} \right] & = \hbar_{\phi } R_{m}^{\phi } \left( \eta \right),L_{\chi } \left[ {\chi_{m} \left( \eta \right) - X_{m} \chi_{m - 1} \left( \eta \right)} \right] = \hbar_{\chi } R_{m}^{\chi } \left( \eta \right), \\ \end{aligned}$$

The corresponding boundary conditions are:

28 $$\begin{aligned} f_{m} \left( 0 \right) & = f_{m}^{\prime \prime \prime } \left( 0 \right) - \gamma f_{m}^{\prime \prime } \left( 0 \right) = \theta_{m} \left( 0 \right) - \beta \theta_{m}^{\prime } \left( 0 \right) = \phi_{m} \left( 0 \right) = \chi_{m} \left( 0 \right) = 0 \\ f_{m}^{\prime } \left( \infty \right) & = \theta_{m} \left( \infty \right) = \phi_{m} \left( \infty \right) = \chi_{m} \left( \infty \right) = 0 \\ \end{aligned}$$

Here

29 $$R_{m}^{f} \left( \eta \right) = f_{m - 1}^{\prime \prime \prime } - f_{m - 1}^{v} - Mf_{m - 1}^{\prime } - \beta_{0} \mathop \sum \limits_{k = 0}^{m - 1} f_{m - 1 - k}^{\prime } f_{k}^{\prime } + \mathop \sum \limits_{k = 0}^{m - 1} f_{m - 1 - k} f_{k}^{\prime \prime } + 1$$

30 $$\begin{aligned} R_{m}^{\theta } \left( \eta \right) & = \left( {1 + Rd} \right)\theta_{m - 1}^{\prime \prime } + \Pr \left( {\mathop \sum \limits_{k = 0}^{m - 1} f_{m - 1 - k} \theta_{k}^{\prime } + Nt\mathop \sum \limits_{k = 0}^{m - 1} \theta_{m - 1 - k}^{\prime } \theta_{k}^{\prime } + Nb\mathop \sum \limits_{k = 0}^{m - 1} \theta_{m - 1 - k}^{\prime } \theta_{k}^{\prime } } \right) \\ & \quad + \mathop \sum \limits_{k = 0}^{m - 1} f_{m - 1 - k}^{\prime \prime } f_{k}^{\prime \prime } + \mathop \sum \limits_{k = 0}^{m - 1}\, f_{m - 1 - k}^{\prime \prime \prime }\, f_{k}^{\prime \prime \prime } , \\ \end{aligned}$$

31 $$R_{m}^{\phi } \left( \eta \right) = \phi_{m - 1}^{\prime \prime } + \frac{Nt}{{Nb}}\theta_{m - 1}^{\prime \prime } + Le\mathop \sum \limits_{k = 0}^{m - 1} f_{m - 1 - k} \phi_{k}^{\prime }$$

32 $$R_{m}^{\chi } \left( \eta \right) = \chi_{m - 1}^{\prime \prime } + Sc\mathop \sum \limits_{k = 0}^{m - 1} f_{m - 1 - k} \chi_{k}^{\prime } - Pe\left( {\sigma \phi_{m - 1}^{\prime \prime } + \mathop \sum \limits_{k = 0}^{m - 1} \chi_{m - 1 - k} \phi_{k}^{\prime \prime } + \mathop \sum \limits_{k = 0}^{m - 1} \chi_{m - 1 - k}^{\prime } \phi_{k}^{\prime } } \right).$$

where

33 $$X_{m} = \left\{ {\begin{array}{*{20}l} {0,} \hfill & {\quad {\text{if}}\,p \le 1} \hfill \\ {1,} \hfill & {\quad {\text{if}}\,p > 1} \hfill \\ \end{array} } \right..$$

## Numerical solution

To get Numerical solution for the Eqs. (8–11) along with boundary conditions (12–13), the procedure as follows. In order to use this method, the higher order ODEs must be transformed into a set of first order ODEs. For this purpose, assume that:

$$\begin{aligned} f & = \Psi (1),\quad f^{{\prime }} = \Psi (2),\quad f^{{{\prime \prime }}} = \Psi (3),\quad f^{{{\prime \prime \prime }}} = \Psi (4),\quad f^{{{\prime \prime \prime \prime }}} = \Psi (5),\quad f^{{{\prime \prime \prime \prime \prime }}} = \Psi^{{\prime }} (5) \\ \theta & = \Psi (6),\quad \theta^{{\prime }} = \Psi (7),\quad \theta^{{{\prime \prime }}} = \Psi^{{\prime }} (7),\quad \phi = \Psi (8),\quad \phi^{{\prime }} = \Psi (9),\quad \phi^{{{\prime \prime }}} = \Psi^{{\prime }} (9) \\ \chi & = \Psi (10),\quad \chi^{{\prime }} = \Psi (11),\quad \chi^{{{\prime \prime }}} = \Psi ^{\prime}(11) \\ \end{aligned}$$

Then the system of equations along with their respective equations can be written as:

34 $$\Psi^{\prime } (5) = \left[ {\frac{{\Psi (4) - M\Psi (2) - \beta_{0} \Psi (2)\Psi (2)^{2} + \Psi (1)\Psi (3) + 1}}{{\lambda_{1} }}} \right]$$

35 $$\Psi^{\prime } (7) = - \left[ {\frac{{\left( \begin{gathered} \Pr \left[ {\Psi (1)\Psi (7) + Nb\Psi (7)\Psi (9) + Nt\Psi (7)^{2} } \right] \hfill \\ + Br\Psi (3)^{2} + \frac{Br}{{\lambda_{1} }}\Psi (4)^{2} + MBr\Psi (2)^{2} \hfill \\ \end{gathered} \right)}}{{\left( {1 + Rd} \right)}}} \right]$$

36 $$\Psi^{\prime } (9) = - \left[ {\frac{Nt}{{Nb}}\Psi ^{\prime}(7) + Le\Psi (1)\Psi (9)} \right]$$

37 $$\Psi^{\prime } (11) = - \left[ {Sc\Psi (1)\Psi (11) - \Pr \left[ {\left( {\sigma + \Psi (10)} \right)\Psi ^{\prime}(9) + \Psi (9)\Psi (11)} \right]} \right]$$

with boundary conditions:

38 $$\begin{aligned} & \Psi_{a} (2) - \gamma \Psi_{a} (3) - \lambda ,\Psi_{a} (1) - S,\Psi_{a} (8) - 1,\Psi_{a} (6) - 1 - \beta \Psi_{a} (7) \\ & \Psi_{b} (2) - 1,\Psi_{a} (10) - 1,\Psi_{b} (10) - 0,\Psi_{b} (8) - 0,\Psi_{b} (6) - 0 \\ \end{aligned}$$

## Validiaons and convergence

**Figure 2:**
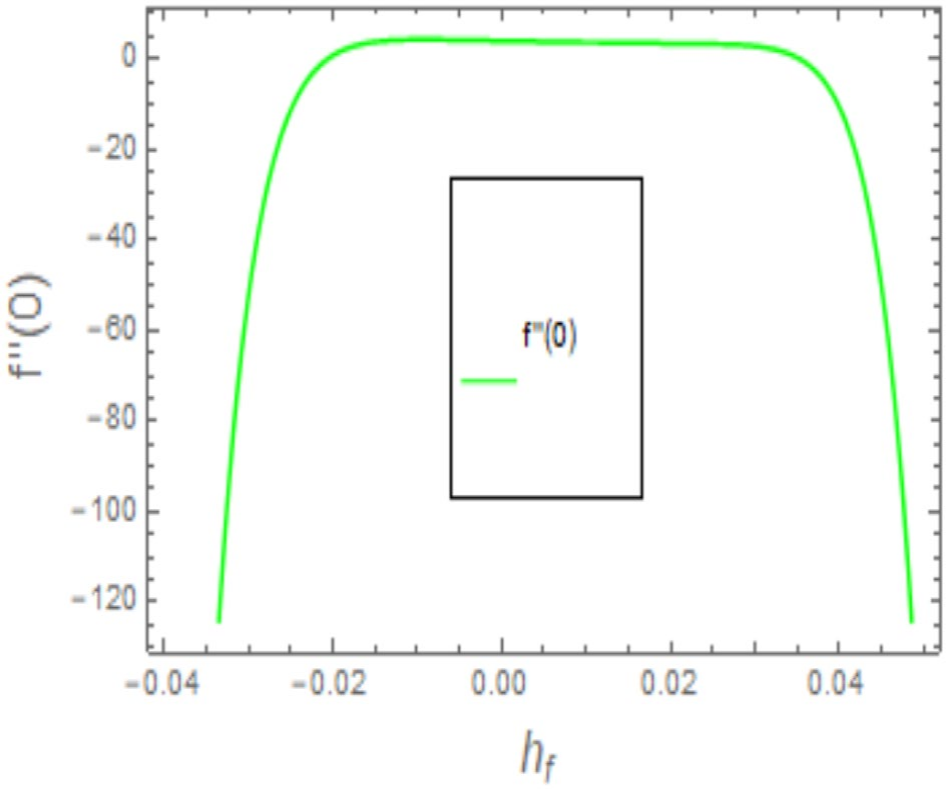
The given figure illustrates a sketch of the h-curve for *f*″(0).

**Figure 3:**
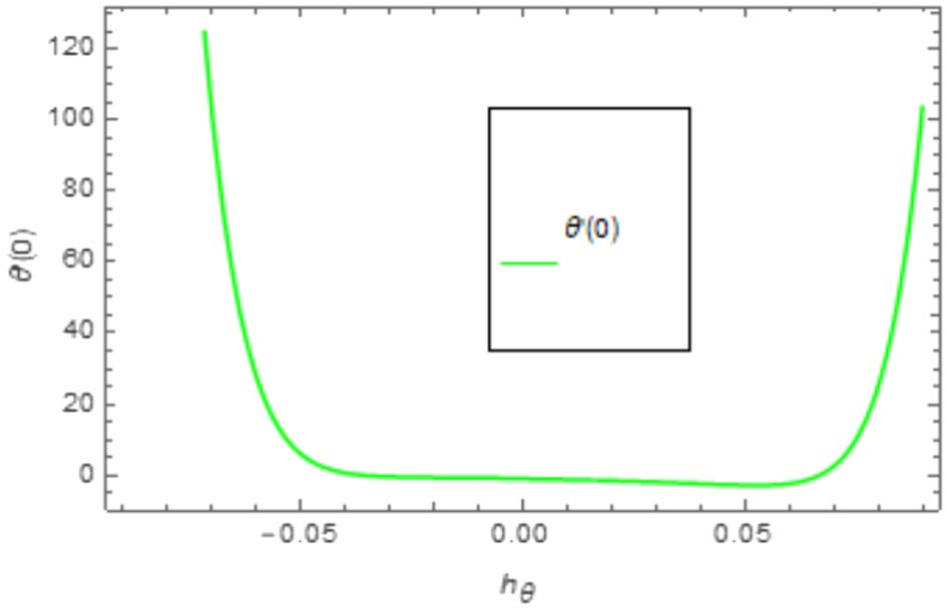
Given figure illustrate sketch of h-curve for *θ*′(0).

**Figure 4:**
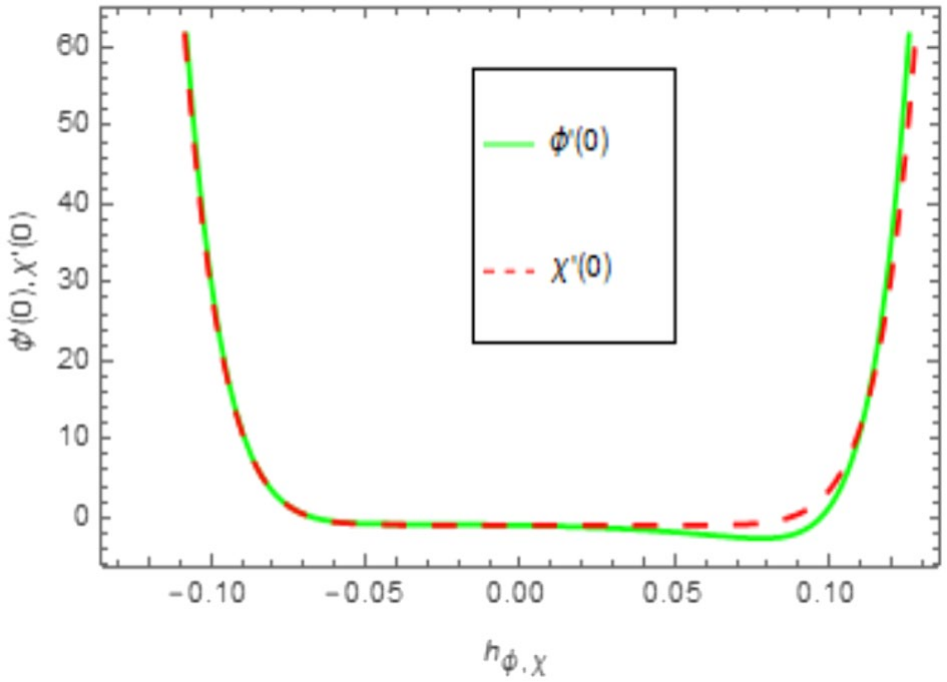
Given figure illustrates a sketch of the h-curve for *ϕ*′(0) and *χ*′(0).

When employing the Homotopy Analysis Method (HAM) to compute series solutions for velocity, temperature, and concentration functions, auxiliary parameters such as *h*_*f*_, *h*_*θ*_, and $h_{\varphi ,x}$ are introduced to ensure the convergence of these solutions. In order to establish the appropriate scope of said parameters, h-curve diagrams have been generated for estimations of $f^{\prime \prime } \left( 0 \right), \theta^{\prime } \left( 0 \right), \phi^{\prime } \left( 0 \right)$, and *χ*′(0) in Figs. 2, 3 and 4, while taking into account diverse values of embedded variables. The h-curves depicted in the figure demonstrate the legitimate areas of convergence. The convergence regions of the h-curves are denoted in Figs. 2, 3, and 4 as − 0.04 ≤ h ≤ 0.4, − 0.05 ≤ h ≤ 0.05, and − 0.10 ≤ h ≤ 0.10, respectively. These ranges are deemed acceptable for the assisting parameters.

**Figure 5:**
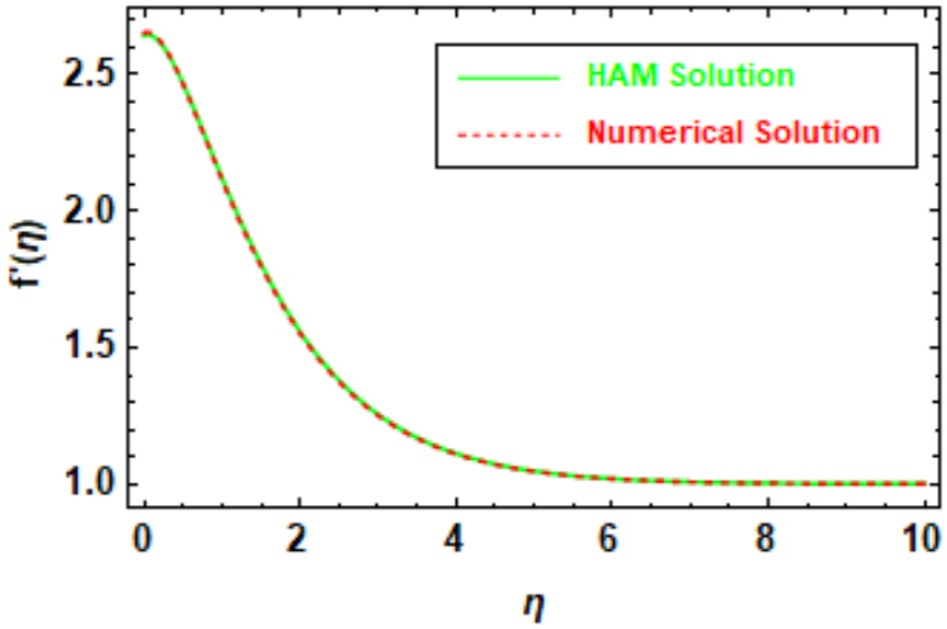
Given figure illustrates the graphical compression of HAM and the numerical solution for *f*′(*η*).

**Figure 6:**
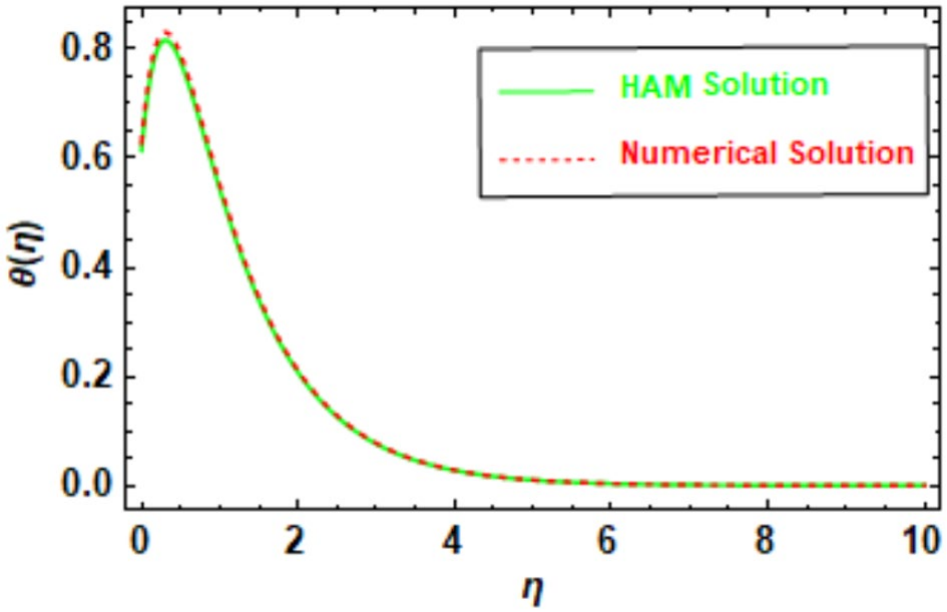
The given figure illustrates the graphical compression of HAM and the numerical solution for *θ*(*η*).

**Figure 7:**
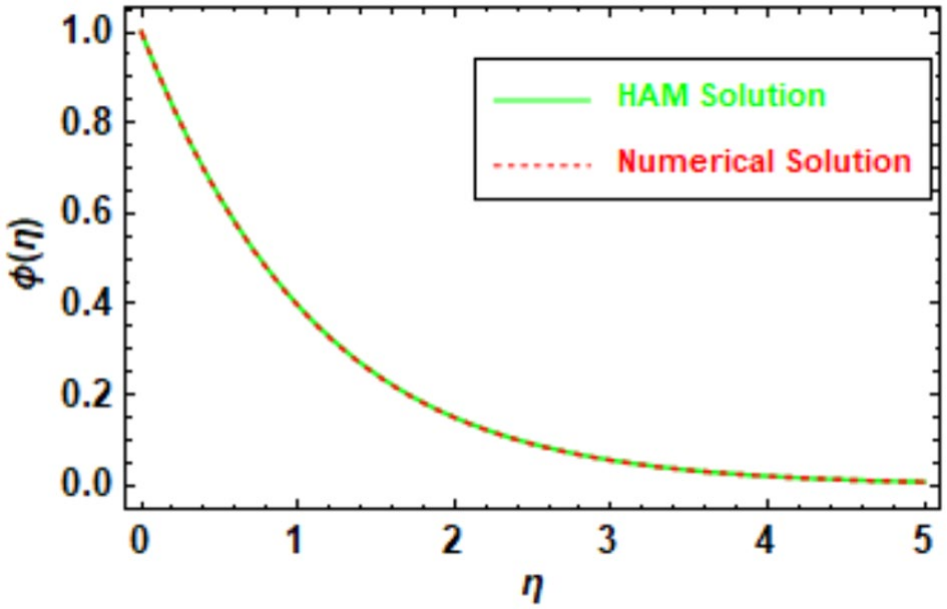
Given figure illustrates the graphical compression of HAM and numerical solution for *ϕ*(*η*).

**Figure 8:**
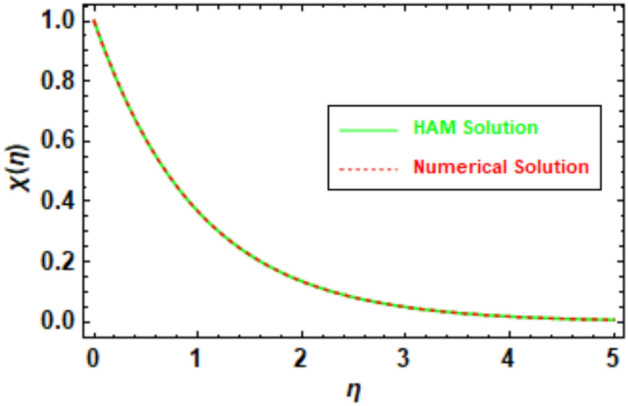
Given figure illustrates the graphical compression of HAM and the numerical solution for *χ*(*η*).

To ensure the accuracy of the obtained results, a rigorous evaluation mechanism was implemented. The Homotopy Analysis Method (HAM) solution was subjected to a meticulous comparison with the solution derived from numerical methods, which demonstrated a remarkable concurrence between the two methodologies. The velocity and temperature graphs of the HAM solution depicted in Figs. 5, 6, 7 and 8 exhibit a congruence with the corresponding graphs of the numerical method solution. In order to augment the accuracy of the HAM solution, a comparative analysis was performed and the outcomes were exhibited in Table 3, which furnishes a comprehensive juxtaposition between the HAM solution and the numerical method solution. Tables 1, 2, 3, and 4, depict the absolute disparity between the HAM solution and the numerical solution across various θ values. The absolute error diminishes with increasing θ. This phenomenon can be attributed to the enhanced precision of the HAM solution as θ attains higher values. Likewise, the numerical solution also improves in accuracy as θ increases, although the HAM solution exhibits a more rapid convergence towards the exact solution compared to the numerical solution. To illustrate, in Table 1, the absolute error for θ = 0 is 0.011199, whereas for θ = 10, it reduces to 0.000013. This disparity reveals that the HAM solutions achieve approximately 9 orders of magnitude higher accuracy for θ = 10 than for θ = 0. Furthermore, the tables provide evidence of the substantial agreement between the HAM solutions and the numerical solutions.

**Table 1:** HAM and numerical comparison *f*′(*η*).

*η*	HAM solution	Numerical solution	Absolute error
0	2.642730	2.653930	0.011199
1	2.120800	2.114150	0.006658
2	1.558500	1.551460	0.007036
3	1.255650	1.251490	0.004167
4	1.112260	1.110180	0.002080
5	1.047990	1.047030	0.000960
6	1.020120	1.019700	0.000424
7	1.008310	1.008130	0.000181
8	1.003390	1.003320	0.000076
9	1.001370	1.001340	0.000031
10	1.000550	1.000540	0.000013

**Table 2:** HAM and numerical comparison *θ*(*η*).

*η*	HAM solution	Numerical solution	Absolute error
0	0.614284	0.628025	0.013741
1	0.542050	0.551113	0.009063
2	0.207424	0.210865	0.003441
3	0.076434	0.077701	0.001268
4	0.028128	0.028595	0.000467
5	0.010350	0.010521	0.000172
6	0.003808	0.003871	0.000063
7	0.001401	0.001424	0.000023
8	0.000515	0.000524	8.553920″ × 10^−6^
9	0.000190	0.000193	3.146830″ × 10^−6^
10	0.000070	0.000071	1.157660″ × 10^−6^

**Table 3:** HAM and numerical comparison *ϕ*(*η*).

η	HAM solution	Numerical solution	Absolute error
0	1.000000	1.000000	0.000000
0.5	0.613241	0.610664	0.002576
1	0.374405	0.370994	0.003412
1.5	0.227972	0.224855	0.003116
2	0.138588	0.136177	0.002411
2.5	0.084171	0.082469	0.001702
3	0.051092	0.049954	0.001138
3.5	0.031003	0.030268	0.000735
4	0.018810	0.018345	0.000465
4.5	0.011410	0.011121	0.000289
5	0.006921	0.006743	0.000179

**Table 4:** HAM and numerical comparison *χ*(*η*).

*η*	HAM solution	Numerical solution	Absolute error
0	1.000000	1.000000	2.220450″ × 10^−16^
0.5	0.605705	0.605751	0.000046
1	0.367012	0.367079	0.000066
1.5	0.222461	0.222526	0.000065
2	0.134877	0.134930	0.000053
2.5	0.081789	0.081827	0.000038
3	0.049601	0.049627	0.000026
3.5	0.030082	0.030099	0.000017
4	0.018245	0.018256	0.000011
4.5	0.011066	0.011073	4.284670″ × 10^−6^
5	0.006712	0.006716	4.284670″ × 10^−6^

## Result and discussion

We have formulated a steady Couple stress MHD water-based bionanofluid across a porous stretching/shrinking sheet in its own plane with impacts of, Bejan number and entropy generation analysis. By applying appropriate similarity transformations, the nonlinear ordinary differential equations, which are derived from the governing nonlinear partial differential equations based on the given assumptions, yield a set of various parameters. An ideal strategy has been employed to achieve the desired results from the modeled parameters. For the solutions, we used semi semi-analytical technique, the Homotopy Analysis Method, and compared it with the numerical technique. In this section, we addressed the effect of various parameters on the velocity, temperature, and nanoparticle concentration profiles depicted in the figures. Graphs are used to show how the impacts of the included parameters are impacted.

### Discussions of the impact of parameters on the velocity profile

**Figure 9:**
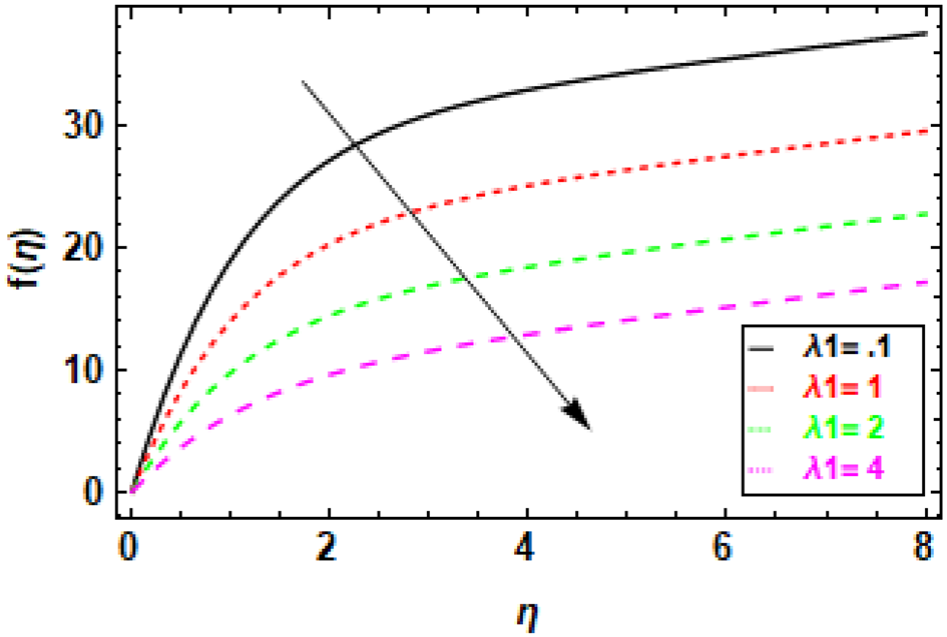
The given figure demonstrates the velocity *f*(*η*) of *λ*_1_ when (a) *h* =  − 0.1, *M* = .2, *β*_0_ = 1, *γ* = 0.3, S = 1.3.

**Figure 10:**
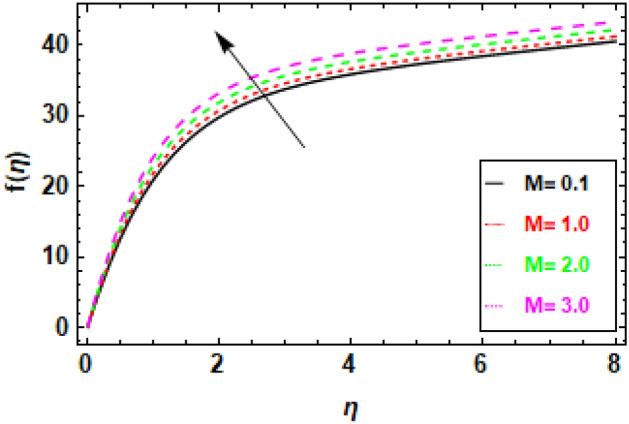
The given figure demonstrates the velocity *f*(*η*) of *M* when (a) *h* =  − .1, *β*_0_ = 1, *λ*_1_ = 0.1, *γ* = 0.3, S = 1.3.

**Figure 11:**
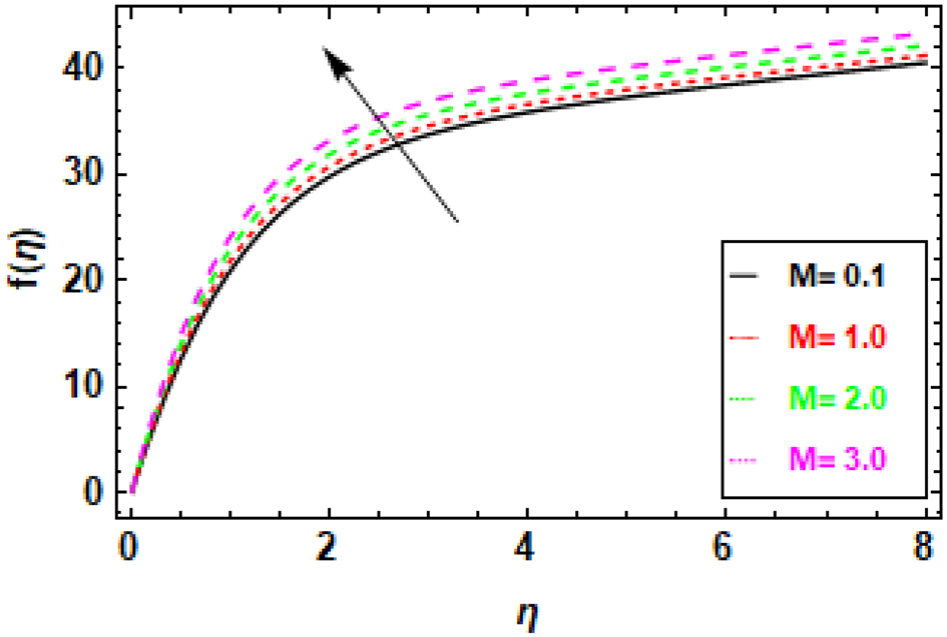
Given figure demonstrates the velocity *f*(*η*) of *β*_0_ when (a) *h* =  − 0.1, *M* = .2, *λ*_1_ = 0.1, *γ* = 0.3, S = 1.3.

**Figure 12:**
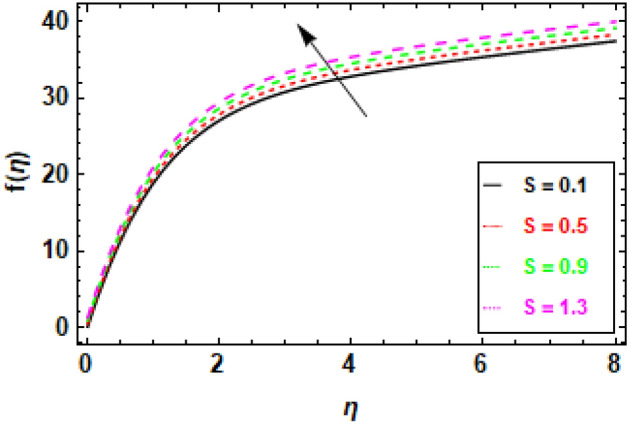
Given figure demonstrates the velocity *f*(*η*) of *S* when (a) *h* =  − 0.1, *M* = .2, *λ*_1_ = 0.1, *β*_0_ = 1, *γ* = 0.3.

**Figure 13:**
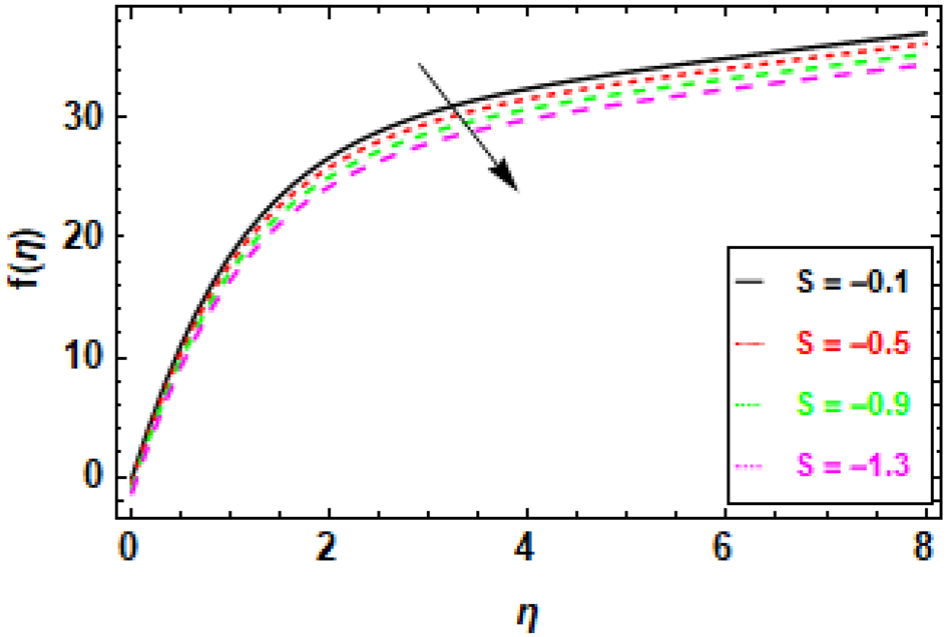
Given figure demonstrates the velocity *f*(*η*) of *S* when (a) *h* =  − 0.1, *M* = .2, *λ*_1_ = 0.1, *β*_0_ = 1, *γ* = 0.3.

Figure 9 depicts the fluctuation in velocity for different values of the Couple stress parameter λ_1_, when the other parameters are held constant. The graphs show a decrease in velocity as the Couple stress parameter λ_1_ rises, illuminating the fact that velocity is negatively impacted by couple stress increases. This is because the Couple stress parameter represents the resistance of the fluid to shear deformation. When the Couple stress parameter is high, the fluid resists shear deformation more strongly. This means that the fluid particles have to move more slowly in order to avoid shear deformation. As a result, the velocity fluctuation decreases. In other words, the higher the couple stress parameter, the more viscous the fluid is. Viscous fluids resist movement, so as the viscosity of the fluid increases, the velocity fluctuation decreases (Fig. 10). The magnetic field’s impacts on the fluid velocity distribution are displayed. The graphic demonstrates that as the charismatic field M grows, the thickness of the boundary layer and the fluid velocity profile diminish. This is due to the Lorentz force, which opposes fluid mobility and so reduces fluid freedom of movement when a magnetic field is present. Consequently, as the magnetic flux rises, the retardation force increases as well, and the resistance supplied to the flow is what reasons the fluid velocity to fall. In Fig. 11 variance in velocity profile, various porosity parameter values are displayed. We see that the fluid has more room to travel and, as a result, its velocity rises as the medium becomes more porous or as *β*_0_ increases. Fluid flow near the boundary layer is slowed by the Darcian body force because it is proportional to the permeability of the medium in the opposite direction. Figure 12, reveals a positive correlation between the velocity profile and the suction parameter S. This correlation suggests that an increment in S results in an enhanced velocity profile. This phenomenon is attributed to the effective removal of fluid from the boundary layer due to the application of suction, which, in turn, reduces the boundary layer's thickness. Consequently, the fluid that remains within the boundary layer must accelerate in order to uphold a consistent mass flow rate. Consequently, there is an augmentation in the velocity profile. The suction parameter, denoted as S, is a dimensionless quantity that quantifies the magnitude of the suction force. In the case where S equals zero, the absence of suction results in a velocity profile that is indistinguishable from the profile that would be observed in the absence of suction. As the parameter S increases, there is an observed increase in the suction velocity, resulting in a narrower and quicker velocity profile. The reduction of the momentum boundary layer due to the augmentation of the suction parameter S might result in various consequences for the flow. As an illustration, the implementation of this technique has the potential to decrease aerodynamic resistance and enhance thermal conductivity. Additionally, this phenomenon has the potential to induce the creation of vortices and various other forms of instability. Figure 13, demonstrates a clear inverse relationship between the injection parameter S and the reduction in velocity fluctuations. Specifically, when S takes on negative values (− 0.1, − 0.5, − 0.9, − 1.3), there is a notable decrease in velocity fluctuations. This phenomenon is attributed to the introduction of fluid into the boundary layer, which results in the stabilization of flow and a decrease in turbulence. As the magnitude of the injection parameter S decreases, there is a corresponding increase in the amount of fluid injected. This increase in injected fluid leads to a further improvement in flow stability. Consequently, there is a marked reduction in the variability of velocity within the system.

### Discussions of the impact of parameters on temperature profile

**Figure 14:**
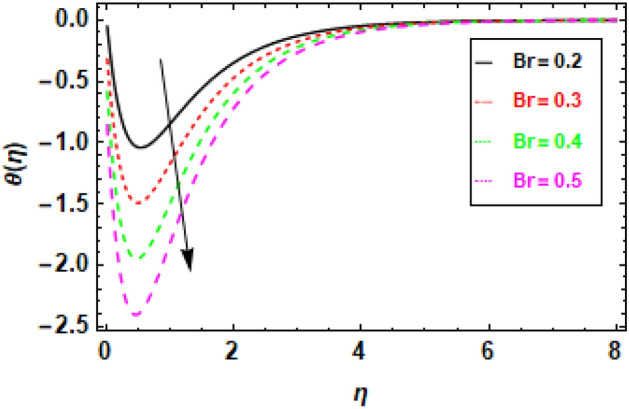
Given figure demonstrates the temperature *θ*(*η*) of Brinkman number Br when (a) *h* = .2, *λ*_1_ = 0.1, *Pr* = .7, *Nb* = .3, *Nt* = .1, *γ* = 0.3.

**Figure 15:**
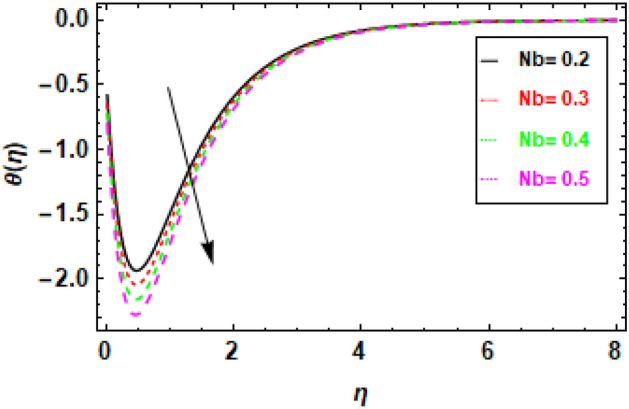
Given figure demonstrates the temperature *θ*(*η*) of Brownian motion parameter Nb. when (a) *h* = .2, *λ*_1_ = 0.1, *Pr* = .7, *Br* = .5, *Nt* = .1, *γ* = 0.3.

**Figure 16:**
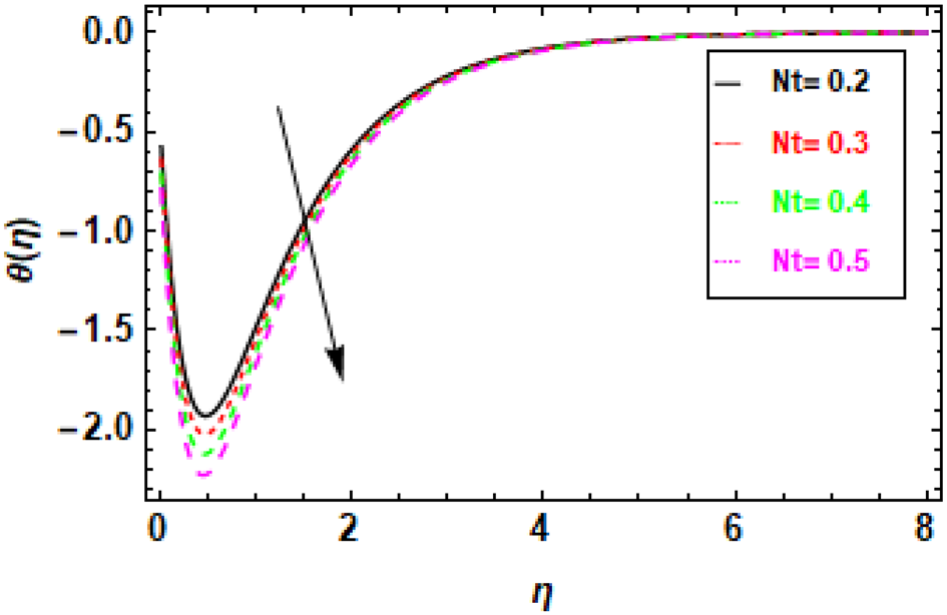
The given figure demonstrates the temperature *θ*(*η*) of (a) thermophoresis parameter Nt when (a) *h* = .2, *λ*_1_ = 0.1, *Pr* = .7, *Nb* = .3, *Br* = .5, *γ* = 0.3.

**Figure 17:**
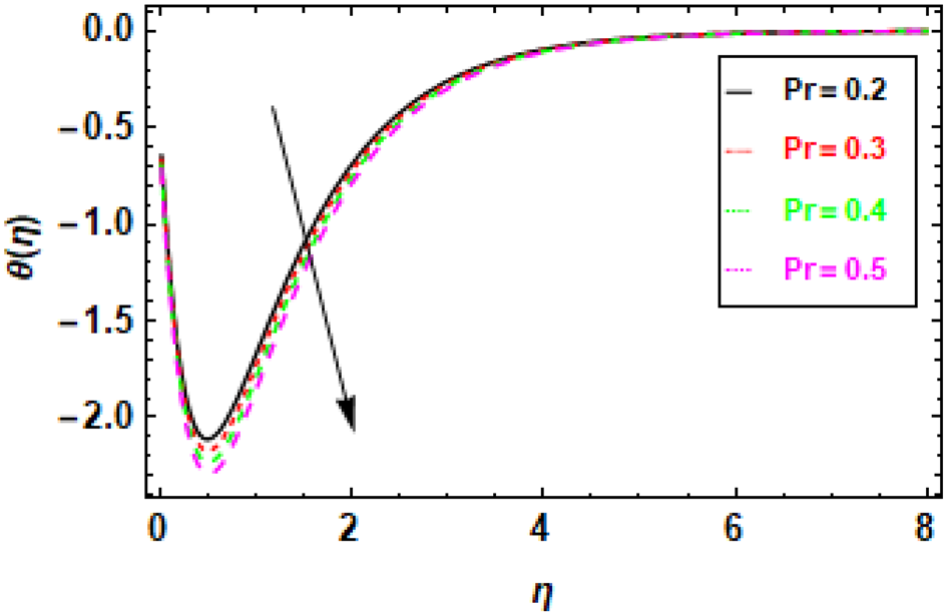
Given figure demonstrates the temperature *θ*(*η*) of Prandtl number Pr. When *h* = .2, *λ*_1_ = 0.1, *Nb* = .3, *Br* = .5, *Nt* = .1, *γ* = 0.3.

Figures 14, 15, 16 and 17 present the temperature distributions corresponding to a variety of Le, br, Nt, and Nb values. These figures provide insights into the impact of the thermophoresis parameter Nt, Lewis number (Le), Brownian motion parameter Nb, and the Prandtl number Pr on the fluid flow's temperature. In the fluid flow region, higher values of Nt and Nb contribute to enhanced thermal conduction. Figure 14 displays the impact of the Lewis number, it is a dimensionless parameter, serves to quantify the relative significance of heat conduction versus mass diffusion. When the Lewis number is higher, it implies a greater influence of heat conduction compared to mass diffusion. Similarly, the Brownian motion parameter measures the impact of Brownian motion on heat transfer, with higher values indicating a stronger dominance of Brownian motion Fig. 15. The thermophoresis parameter quantifies the influence of thermophoresis on the heat transfer phenomenon, with a greater thermophoresis value indicating a stronger dominance of thermophoresis, this effect is shown in Fig. 16. The findings shown in the figures indicate a positive correlation between the temperature and the thermophoresis parameter (Nt) as well as the Brownian motion parameter (Nb). This occurrence arises as a result of the synergy between thermophoresis and Brownian motion. Together, they promote and facilitate the efficient transfer of thermal energy, enabling the flow of heat from higher temperature regions to lower temperature regions. Nevertheless, the impact of thermophoresis is more significant in comparison to the influence of Brownian motion. The data presented in Fig. 17 indicates that an elevation in the Prandtl number leads to a significant reduction in temperature readings along the boundary layer that is orthogonal to the plate's surface. The Prandtl number is a nondimensional parameter that delineates the relationship between thermal diffusivity and momentum diffusivity in the boundary layer regime. The Prandtl number serves as a metric for comparing the relative magnitudes of momentum diffusivity and thermal diffusivity in a fluid. A larger Prandtl number signifies a greater predominance of momentum diffusivity.The quantity mentioned above represents the ratio between the thermal conductivity of a fluid and the product of its specific heat capacity and dynamic viscosity. There has been a notable reduction in the magnitude of the thermal boundary layer depth. The Prandtl number (Pr) exhibits an inverse relationship with the efficiency of the heat transfer process so an increase in Pr leads to a decrease in efficiency. The inverse holds true in the case of the Prandtl number (Pr). As the Prandtl number exhibits an increase, the temperature gradient within the fluid flow has a tendency towards a more uniform distribution, hence implying a decrease in the efficiency of heat conduction. This phenomenon can be attributed to the fact that a higher Prandtl number signifies a greater prominence of momentum diffusivity relative to thermal diffusivity, resulting in less effective heat transmission. In general, the data presented indicates that the incorporation of the thermophoresis parameter (Nt) and the Brownian motion parameter (Nb) can enhance the efficiency of heat transfer within a fluid flow. Nevertheless, the impact of thermophoresis is more significant in comparison to the influence of Brownian motion.

### Discussions of the impact of parameters on concentration profile

**Figure 18:**
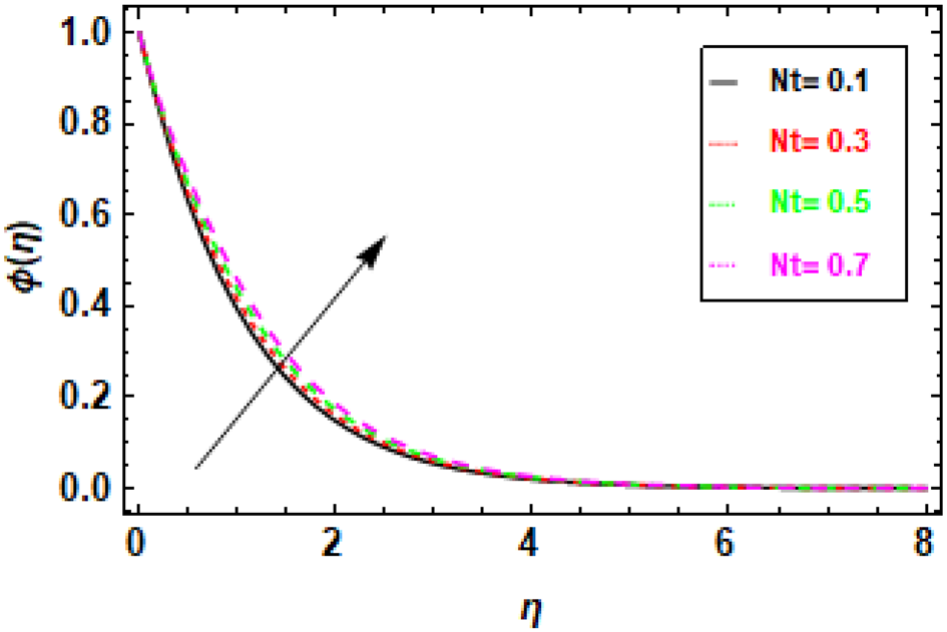
Given figure demonstrates the concentration profiles for different values of thermophoresis parameter Nt. when *h* = .2, *Nb* = .3, *Le* = 0.1, *γ* = 0.3.

**Figure 19:**
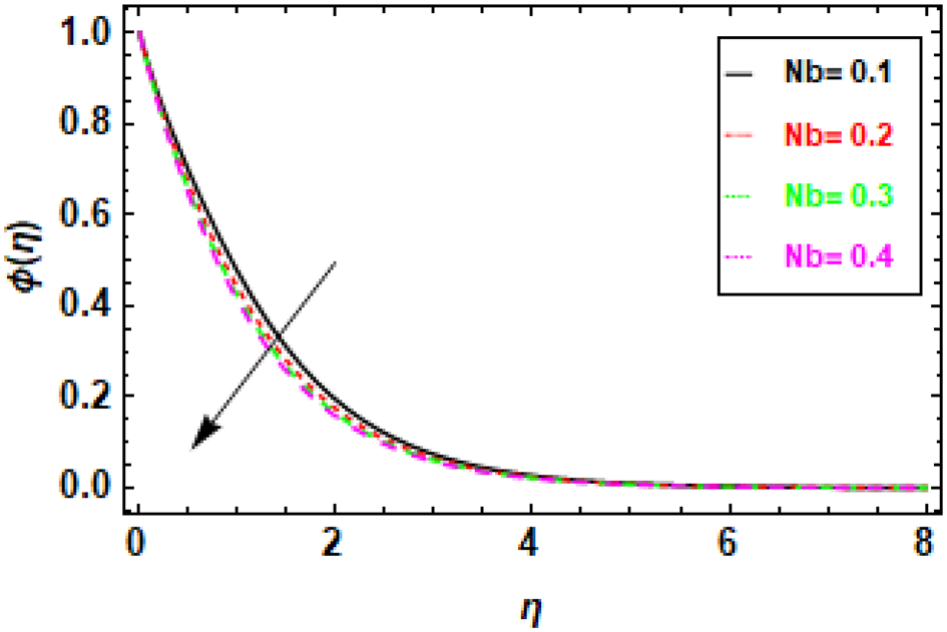
The concentration profiles for various values of the Brownian motion parameter (Nb) are illustrated in the provided figure. When *h* = .2, *Nt* = .4, *Le* = 0.1, *γ* = 0.3.

**Figure 20:**
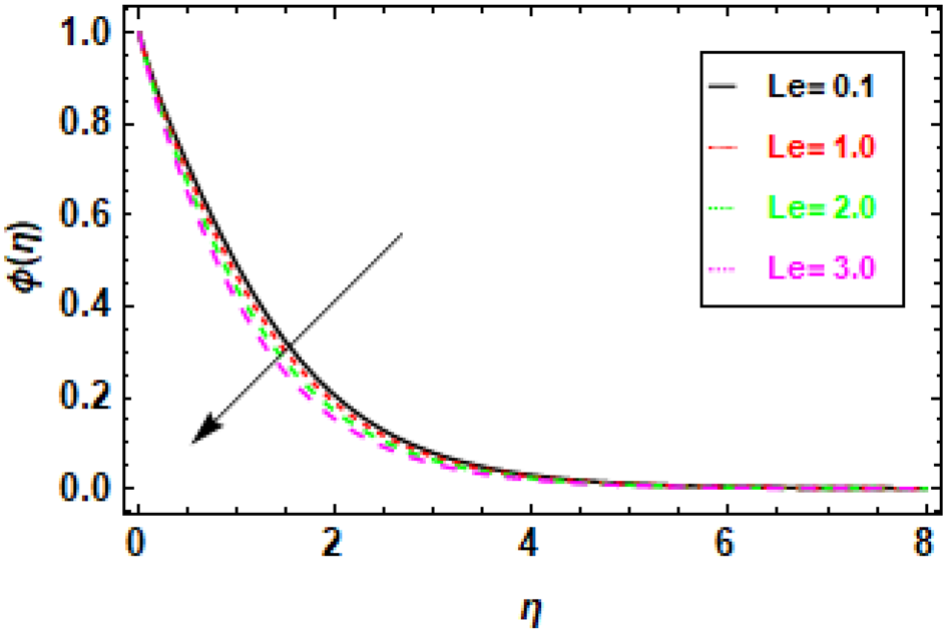
The figure illustrates concentration profiles corresponding to various values of the Lewis number (Le). When *h* = .2, *Nb* = .3, *Nt* = .4, *γ* = 0.3.

In Fig. 18, we observe a direct relationship between the Brownian motion parameter Nb and the concentration profile. Specifically, as Nb increases, the concentration also increases. Meanwhile, Fig. 19 provides insights into the influence of the thermophoresis parameter Nt on the flow's concentration profiles. It is well-established that an increase in Nt leads to a reduction in the concentration profiles of the flow. In Fig. 18, we can observe the relationship between the concentration profile and the Brownian motion parameter Nb. It illustrates that an increase in Nb leads to a corresponding increase in the concentration profile. Brownian motion results from the random movement of particles driven by their interactions with nearby molecules. A higher value of the Brownian motion parameter signifies a more pronounced influence of Brownian motion, which, in turn, raises the likelihood of particle collisions and the subsequent formation of clusters. As a result, these clusters exhibit a higher concentration of particles compared to the surrounding fluid. Figure 19 depicts the negative correlation between the concentration profile and the thermophoresis parameter Nt, suggesting that an increase in Nt leads to a drop in the concentration profile. Thermophoresis is the term used to describe the process in which particles experience displacement due to the presence of a temperature difference. A higher thermophoresis value indicates a stronger thermophoretic effect, leading to a greater probability of particle movement towards the colder region of the fluid. Consequently, the concentration of particles in the colder zone will exhibit a reduction, whereas the concentration of particles in the warmer region will experience an increase. In Fig. 20, we can observe how changes in the Lewis number (Le) affect concentration profiles. The graphical depiction makes it evident that an increase in Le leads to a decrease in concentration. The Lewis number is a parameter that signifies the relationship between the diffusivity of thermal energy and the diffusivity of mass species within the realm of nano-particles. When Le equals 1, it signifies that the thermal diffusivity of the nanofluid is equivalent to the species diffusivity of the nanoparticles. Additionally, the boundary layers' thicknesses are also equivalent. When the Lewis number is less than or equal to one, the diffusivity of mass surpasses the diffusivity of heat. Conversely, when the Lewis number is greater than one, the diffusivity of heat is greater than the diffusivity of mass.

### Discussions of the impact of parameters on Motile organism profile

**Figure 21:**
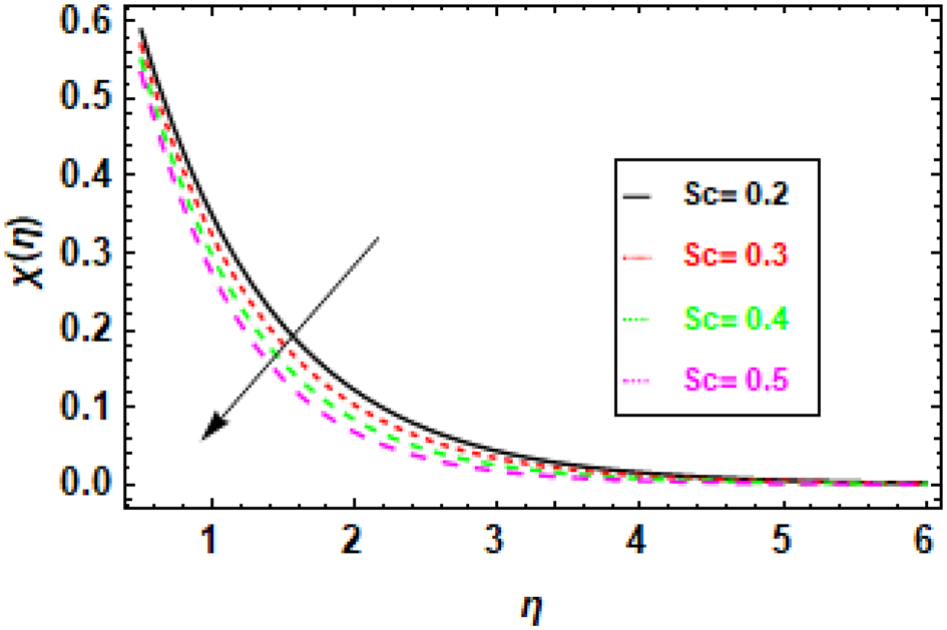
The figure illustrates microorganism profiles corresponding to various values of the Schmidt number (Sc). When *h* = .2, *Pe* = 1, *σ* = .1, *γ* = 0.3.

**Figure 22:**
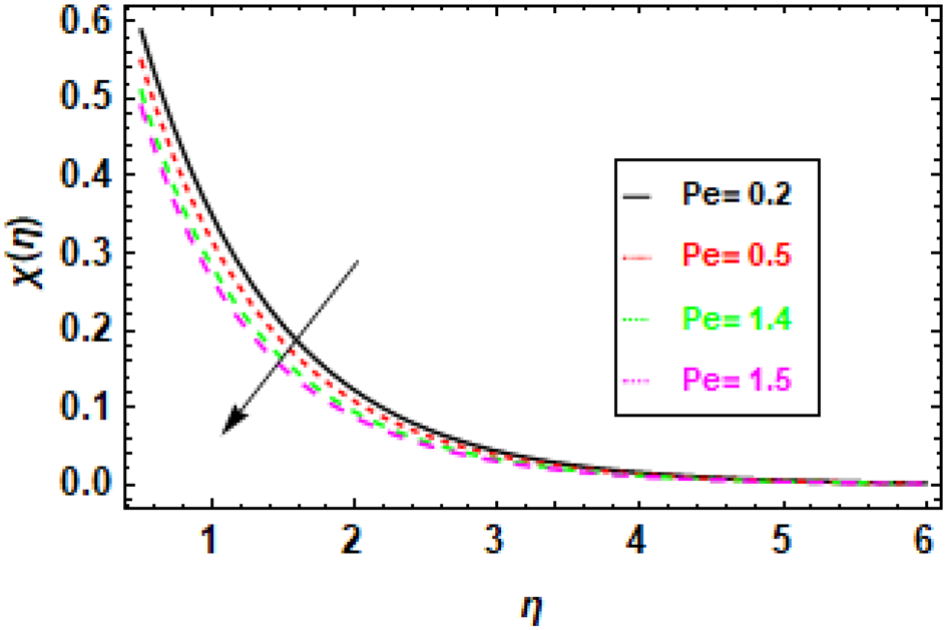
The figure illustrates microorganism profiles corresponding to various values of the Péclet number (Pe). When *h* = .2, *Sc* = 0.2, *σ* = .1, *γ* = 0.3.

Figure 21, shows that when Schmidt number (Sc) increases, the concentration of microorganisms in the fluid decreases, demonstrating a negative association between these two variables. The behavior seen can be explained by considering the relative importance of mass diffusion and momentum diffusion. Because mass diffusion is more prominent as the Schmidt number rises, microorganisms have more time to diffuse away from the surface before being carried by the fluid flow. This results in a lower concentration of microorganisms in the fluid. Figure 22, demonstrates an inverse relationship between the Péclet number (Pe) and the concentration of microorganisms in the fluid. Specifically, an increase in Pe leads to a decrease in microorganism concentration, while a decrease in Pe results in an increase in microorganism concentration. This is because the Péclet number quantifies how much more important advection is than diffusion when it comes to the transfer of compounds by fluid flow, as stated above. A higher Péclet number indicates that advection is more dominant than diffusion, increasing the likelihood that microorganisms are carried by the fluid flow before diffusing away from the surface. The illustration shows that the profiles of microorganisms are not perfectly symmetrical. Microorganisms have a greater chance of being transported away from the surface and along the direction of the fluid flow, which explains this phenomenon. This occurs because the fluid's velocity is highest in the direction of flow.

**Figure 23:**
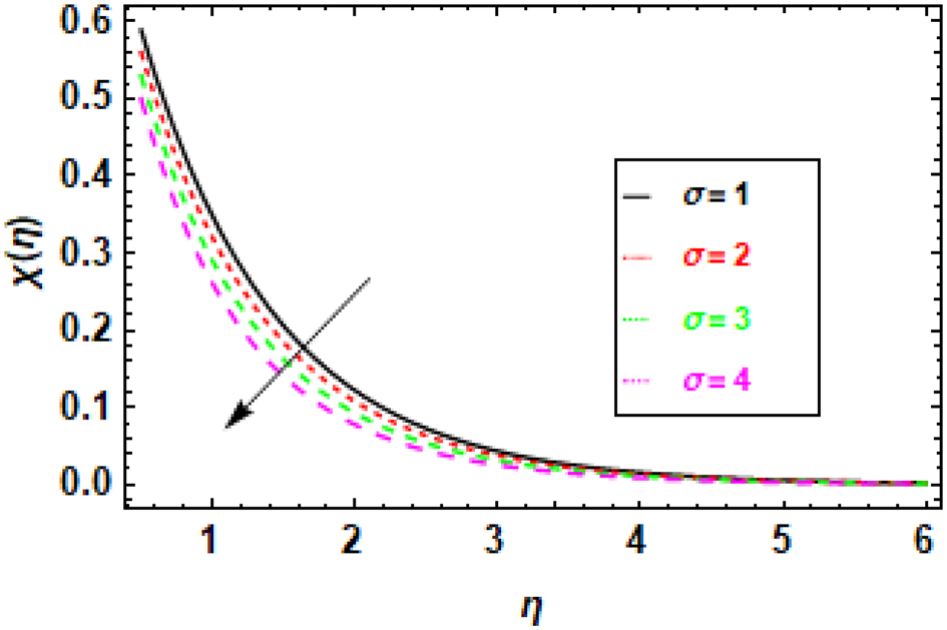
The figure illustrates microorganism profiles corresponding to various values of the Dimensionless constant (**σ**). When *h* = .2, *Sc* = 0.2, *Pe* = 1, *γ* = 0.3.

The preceding graph can be used to learn how the Péclet number affects the distribution of bacteria in a liquid. The aforementioned information can be used to better design bioreactors and other devices used in microorganism growth. Figure 23, illustrates the correlation between the microbe concentration in a fluid and the dimensionless constant, showing that an increase in the latter causes a commensurate change in the former. The dimensionless parameter σ serves as an indicator of the significance of thermophoresis, which refers to the transport of particles by temperature gradients, in comparison to diffusion. A greater value of σ indicates a greater significance of thermophoresis relative to diffusion, thereby increasing the likelihood of microorganisms being conveyed away from the surface through temperature gradients before their diffusive escape. The presented data indicate an inverse relationship between the concentration of microorganisms and the value of σ, where an increase in the latter results in a decrease in the former. The reason behind the accelerated transportation of microorganisms away from the surface is attributed to the heightened thermophoretic effect, which is directly proportional to the increase in. The presented figure indicates that the profiles of microorganisms exhibit asymmetry. This phenomenon occurs due to the propensity of microorganisms to be carried away from the surface in the direction of the temperature gradient. This phenomenon occurs due to the elevated temperature gradient in the direction of the gradient. The aforementioned diagram facilitates comprehension of the impact of the non-dimensional constant σ on the dispersion of microorganisms within a fluid medium. The aforementioned data can be utilized in the development of bioreactors and other apparatuses employed for the cultivation of microorganisms.

### Discussions of the impact of parameters on entropy profile

**Figure 24:**
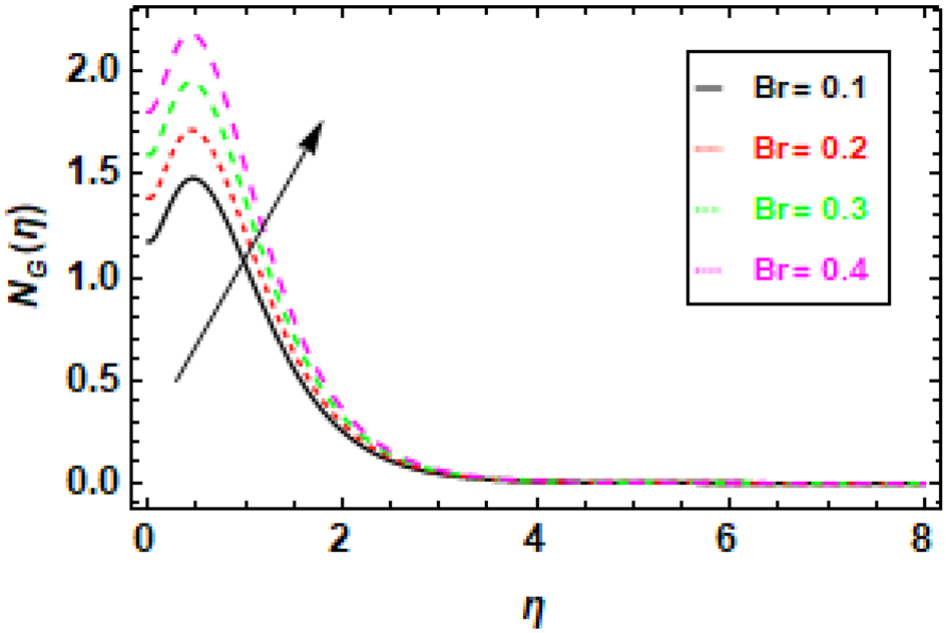
This image shows how the entropy varies throughout a range of Brinkman numbers Br.

**Figure 25:**
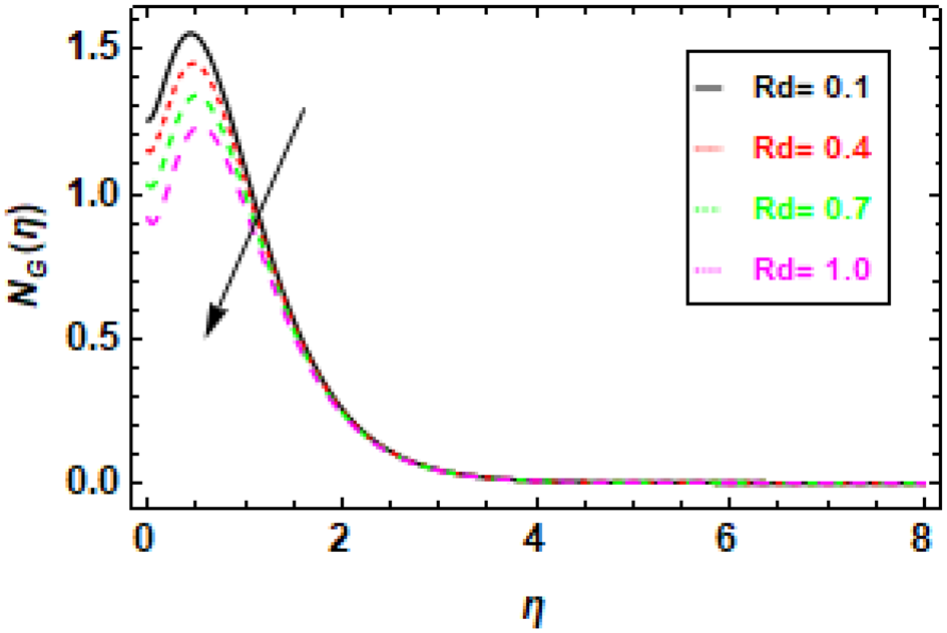
The figure demonstrates how entropy varies with different values of the radiation parameter Rd.

**Figure 26:**
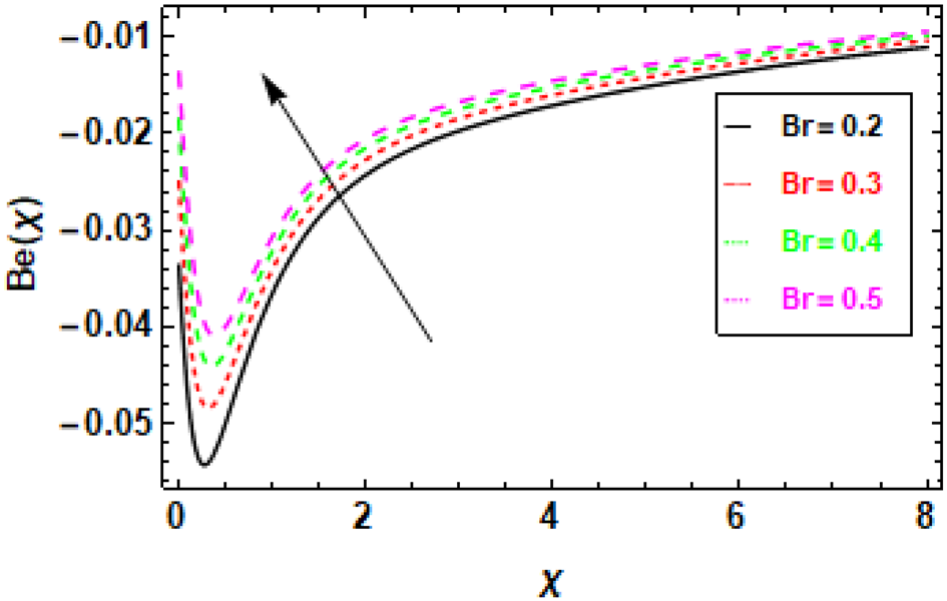
The aforementioned figure illustrates the variation of the Bejan number in response to alterations in the Brinkman number, denoted as *Br*.

**Figure 27:**
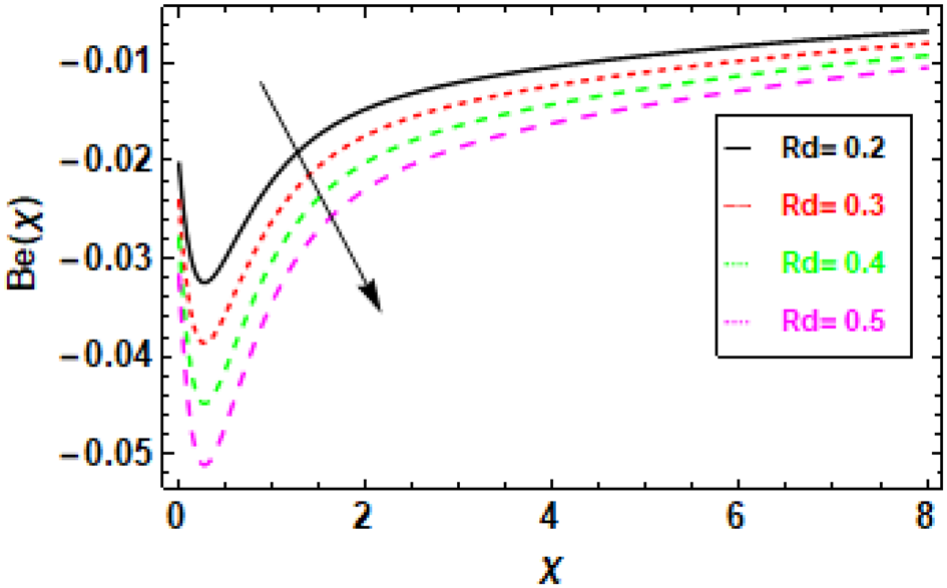
Given figure illustrates the fluctuation in Bejan number concerning various values of the radiation parameter, denoted as *Rd*.

**Figure 28:**
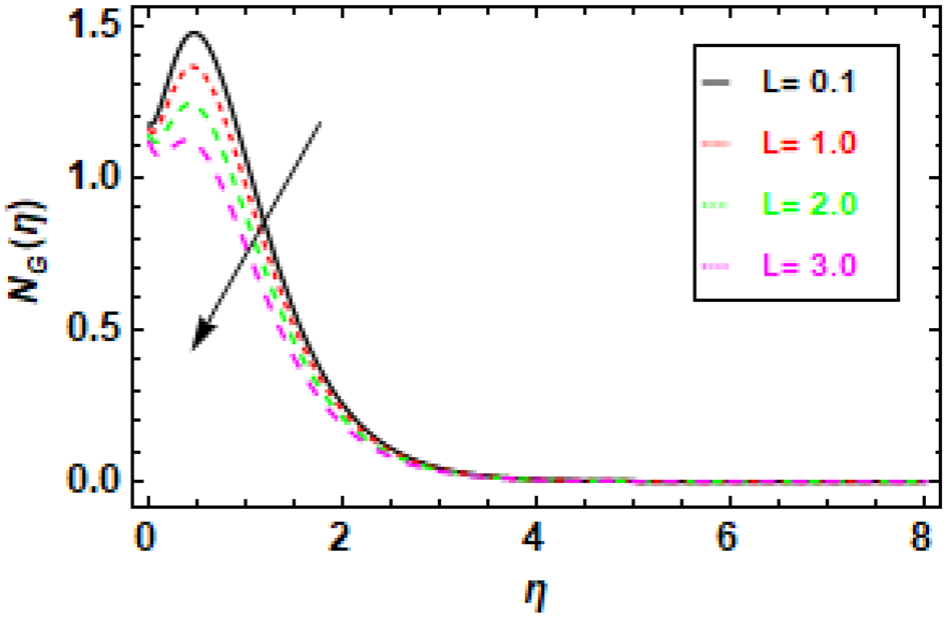
Given figure illustrates the changes in entropy across different values of the diffusion parameter (L).

Figure 24 illustrates the impact of entropy generation on various Brinkman numbers (Br). The graph illustrates the impact of different Brinkman numbers on the entropy generation profile, indicating a marginal rise in the entropy formation discrepancy as Br values are altered. The aforementioned occurrence can be ascribed to the Brinkman number, which functions as a thermal stimulus, instigating the production of heat within the movable fluid particle layers. The production of entropy in the flow channel is stimulated by the generation of heat and its transmission from the heat wall. Suitable control of the Brinkman number is necessary to achieve a reduction in entropy. At the surface, entropy generation is fairly common. In Fig. 25, it is depicted how the creation of entropy has changed for many values of the radiation parameter Rd. The figure led us to conclude that different values of Rd result in a minor reduction in the entropy generation profile’s variability. As the value of the radiation parameter increases, there is an observed increase in the amount of heat transported through the process of thermal radiation. The process of heat transfer being discussed exhibits a higher degree of uniformity compared to heat transfer through viscous dissipation. This implies that the distribution of entropy creation is more uniform across the system, leading to a decrease in the variability of the entropy generation profile. The graphical representation of the correlation between the Bejan number (Be) and the Brinkman number (Br) is illustrated in Fig. 26. The available empirical data indicates that there exists an inverse relationship between the Brinkman number and the Bejan number. The rationale behind this is that the Brinkman number serves as a metric for assessing the relative significance of viscous dissipation and diffusion inside a porous material. As the Brinkman number exhibits an increase, the significance of viscous dissipation surpasses that of diffusion, resulting in an escalation of the entropy generation rate. Consequently, there is a resultant reduction in the Bejan number. Heat generation inside fluid particles in motion generally happens in the inner layers due to their proximity to the hot surface and higher velocity. The increase in entropy of the paraboloid of revolution flow can be attributed to the thermal energy generated and transferred from the heated surface. The rationale behind this phenomenon stems from the concept that the entropy of a given system serves as a quantitative indicator of its level of disorder. Consequently, the process of heat transfer contributes to an increase in the disorderliness of said system. To mitigate entropy, it is imperative to regulate the Brinkman number. This can be accomplished by augmenting the diffusion process, which can be attained by enhancing the porosity of the medium. Porosity refers to the proportion of void volume in relation to the overall volume of a given medium. An increased porosity corresponds to a greater volume of available space for flow, hence facilitating enhanced diffusion. Figure 28 shows that a reduction in the radiation parameter (Rd) led to a decrease in the Bejan number. This is because the radiation parameter measures the importance of radiation heat transfer relative to conduction heat transfer. As the radiation parameter decreases, conduction heat transfer becomes more important, which leads to a decrease in the entropy generation rate. This, in turn, leads to an increase in the Bejan number. The decline in temperature gradients is attributed to the increase in the radiation parameter Rd. This is because radiation heat transfer is a volumetric process, meaning that it occurs throughout the entire fluid. Conduction heat transfer, on the other hand, is a surface process, meaning that it only occurs at the boundaries between the fluid and the solid walls. As the radiation parameter increases, the amount of radiation heat transfer increases, which helps to reduce the temperature gradients in the fluid. In summary, Fig. 27 shows that the Bejan number is inversely proportional to the radiation parameter. This is because radiation heat transfer is a more efficient way of transferring heat than conduction heat transfer. As the amount of radiation heat transfer increases, the entropy generation rate decreases, which leads to an increase in the Bejan number.

**Figure 29:**
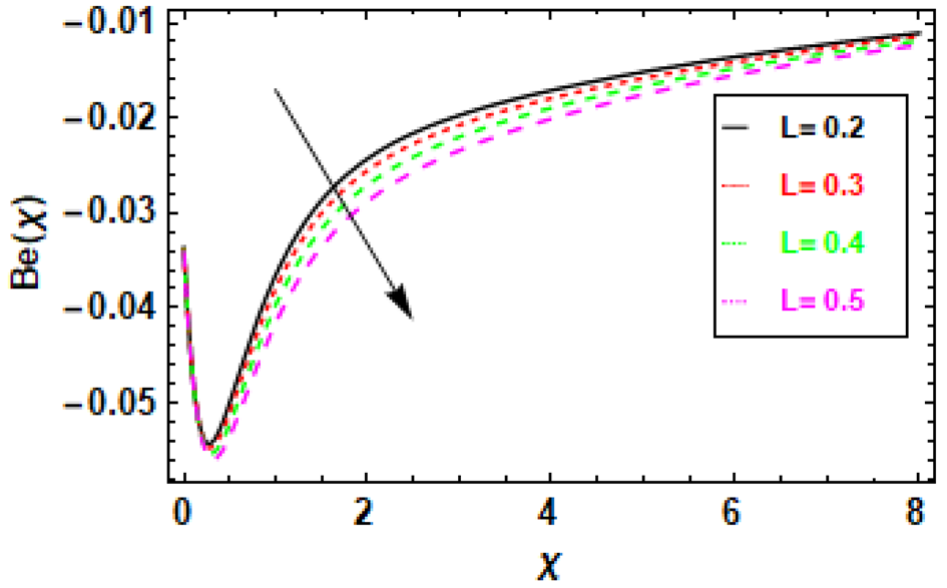
The figure shows how the Bejan number changes as L, the diffusion parameter, is varied.

Figures 28 and 29 illustrate how the diffusion parameter (L) affects entropy generation and the Bejan number. As the estimation value (L) increases, both entropy generation and the Bejan number also increase. Bejan number increases because, as diffusion increases, mass transfer irreversibility has a more significant effect than viscous dissipation irreversibility.

## Numarical discussions on physical quantities

Table 5, presents the influence of magnetic, porosity, and Couple fluid parameter on the Skin friction coefficient (*C*_*fx*_). The findings in Table 5 indicate a clear association between increasing parameter values and heightened skin friction. Physically, the escalating magnitude of these parameters engenders supplementary friction forces, consequently leading to an enhancement in skin friction. For instance, augmenting the magnetic parameter, such as amplifying the strength of the magnetic field, may potentially result in a decrease in skin friction. In Magnetohydrodynamic (MHD) flow, the magnetic field exerts a stabilizing effect on fluid motion, inhibiting turbulence, a significant contributor to skin friction. When a magnetic field interacts with an electrically conducting fluid, it induces a Lorentz force that acts upon the fluid, promoting a more orderly flow pattern and dampening fluid motion. This suppression of turbulence diminishes energy dissipation and mixing within the fluid, thereby reducing skin friction. Moreover, elevating the porosity parameter leads to the fluid encountering an increased number of obstacles or voids within the porous medium. These obstacles impede fluid flow, resulting in heightened flow resistance. The greater presence of void spaces creates additional fluid–solid interaction surfaces, contributing to an elevation in skin friction. Furthermore, an increment in the fluid parameter also leads to an upsurge in the skin friction coefficient. Couple stress fluids, unlike Newtonian fluids, exhibit heightened drag when flowing over a surface due to the presence of Couple stresses. Consequently, a higher Couple stress fluid parameter corresponds to a greater skin friction coefficient. In other words, an increased prevalence of couple stresses in the fluid results in augmented drag as it flows over a surface. In conclusion, the results presented in Table 5 underscore the intricate relationship between magnetic, porosity, and couple fluid parameters, and their consequential impact on skin friction. Understanding these relationships is crucial for comprehending the complex dynamics involved in fluid-surface interactions and frictional behavior. Table 6, illustrates that the Nusselt number rises as the Local Reynolds number, Prandtl number, and thermophoretic coefficient rise. This is because a high Local Reynolds number indicates that the fluid is flowing faster, a high Prandtl number shows that the fluid is more viscous, and a positive thermophoretic coefficient implies that particles will gravitate towards the warmer region. As the Brownian motion coefficient and Brinkman number increase, the Nusselt number decreases. This is because a high Brownian motion coefficient implies that the particles will be more dispersed, whereas a high Brinkman number indicates that the particles will be more prone to become trapped in vortices. The Couple stress parameter does not affect the Nusselt number. This is because the Couple stress parameter only impacts momentum transmission and not heat transfer. In summary, the Nusselt number grows with rising Local Reynolds number, Prandtl number, and thermophoretic coefficient and drops with increasing Brownian motion coefficient and Brinkman number. The Couple stress parameter has no effect on the Nusselt number. Table 7 displays the relationship between the Lewis number (Le), thermophoretic coefficient (Nt), Brownian motion coefficient (Nb), and the Sherwood number (*Sh*_*x*_), which quantifies the rate of mass transfer between a fluid and a solid surface. Notably, an increase in the Brownian motion coefficient (Nb) leads to a decrease in the Sherwood number.Conversely, the Lewis number (Le) and thermophoretic coefficient (Nt) demonstrate an increase with rising values of Nb. This phenomenon can be attributed to the behavior of particles, which tend to migrate towards warmer regions when both their thermophoretic coefficient and Lewis number are elevated. However, when the Brownian motion coefficient assumes a larger value, particles exhibit more random movement, subsequently decelerating the rate of mass transfer. Table 8 provides a comprehensive overview of the dynamic patterns observed in the mean concentration of microorganisms (*Nn*_*x*_) within a fluid, under the influence of distinct Schmidt number (Sc) and Peclet number (Pe) values. The Schmidt number, a dimensionless parameter characterizing the ratio between momentum kinematic viscosity and mass diffusivity, plays a vital role in understanding the balance between momentum and mass transport. Notably, a higher Schmidt number suggests a significant predominance of momentum transport over mass transport. Similarly, the Peclet number, another dimensionless parameter, quantifies the relationship between characteristic velocity multiplied by characteristic length and molecular diffusivity. A high Peclet number indicates that advection significantly surpasses the influence of diffusion. Analyzing the data presented in the table, it becomes apparent that there exists a positive correlation between the mean concentration of microorganisms and both the Schmidt number and Peclet number. This correlation arises due to the enhanced transport of microorganisms facilitated by higher Schmidt and Peclet numbers, consequently leading to an increased mean concentration of microorganisms within the fluid. Furthermore, the data in the table provides evidence that variations in the Reynolds number (Re) do not affect the mean concentration of microorganisms. The influence of the Reynolds number is restricted solely to the rate of momentum transfer, whereas the mean concentration of microorganisms solely represents the average concentration of microorganisms present in the fluid. Table 9 deciphering the Nomenclature.

**Table 5:** Skin friction coefficient (*C*_*fx*_) varies for different values of magnetic, porosity, and a couple fluid parameters.

Skin friction coefficient (*C*_*fx*_)	*M*	*β*_0_	*λ*_1_	$Re_{x}^{1/2} C_{f}$
1	0.1			− 3.30024
2	0.2			− 3.36111
3	0.3			− 3.421923
4		0.1		− 3.30024
5		0.2		− 3.36111
6		0.3		− 3.42193
7			0.1	− 3.30024
8			0.2	− 3.48605
9			0.3	− 3.69674

**Table 6:** Variation of Nusselt number (*Nu*_*x*_) for numerous values of local Reynold (Rd), Prandtl (*Pr*), Brownian motion (*Nb*), Thermophoresis (*Nt*), Brinkman (*Br*), and couple stress (*λ*_1_) fluid parameter values.

Nusselt number (*Nu*_*x*_)	*Rd*	*Pr*	*Nb*	*Nt*	*Br*	*λ*_1_	$Re_{x}^{ - 1/2} N_{u}$
1	0.1						1.00376
2	0.2						0.999196
3	0.3						0.994663
4		0.1					1.00376
5		0.2					1.00767
6		0.3					1.01158
7			0.1				1.00376
8			0.2				1.00338
9			0.3				1.00299
10				0.1			1.00376
11				0.2			1.00342
12				0.3			1.00308
13					0.1		1.00376
14					0.2		1.15303
15					0.3		1.3023
16						0.1	1.00376
17						0.2	0.853419
18						0.3	0.836191

**Table 7:** Variation of Sherwood number (*Sh*_*x*_) for various values of Lewis (*Le*), Thermophoresis (*Nt*), and Brownian motion (*Nb*) parameter values.

Sherwood number (*Sh*_*x*_	*Le*	*Nt*	*Nb*	$Re_{x}^{ - 1/2} S_{h}$
1	0.1			0.883889
2	0.2			0.88672
3	0.3			0.889551
4		0.1		0.883889
5		0.2		0.829418
6		0.3		0.774976
7			0.1	0.883889
8			0.2	0.952335
9			0.3	0.920233

**Table 8:** Variation of microorganisms number (*Nn*_*x*_) for various values of Schmidt number (*Sc*), and Peclet number (*Pe*).

Microorganisms number (*Nn*_*x*_)	*Sc*	*Pe*	$Re_{x}^{ - 1/2} Nn_{x}$
1	0.1		0.949363
2	0.2		0.952199
3	0.3		0.955036
4		0.1	0.949363
5		0.2	0.960346
6		0.3	0.97134

**Table 9:** Deciphering the nomenclature.

Symbol/name	Description	Symbol/name	Description
*Re*_*x*_	Local Reynold number (m^2^/s)	*Nb*	Brownian motion factor (dimensionless)
*Sc*	Schmidt number (m^2^/s)	*Nt*	Thermophoresis parameter (dimensionless)
*Sh*_*x*_	Local number for Sherwood (m/s)	*Nn*_*x*_	Density of actively moving microorganisms in a specific area
*u*_*w*_	Velocity near the surface (m/s)	*Nu*_*x*_	Local Nusselt number (dimensionless)
*D*_1_	Thermal slip factor (dimensionless)	*λ*_1_	Couple stress fluid (dimensionless)
*β*_0_	Porosity parameter (dimensionless)	B_0_	Magnetohydrodynamics (MHD) (dimensionless)
*D*_*B*_	Brownian motion coefficient (m^2^/s)	*D*_*n*_	Microorganism diffusivity (m^2^/s)
*D*_*T*_	Temperature-dependent diffusion coefficient	*F*	Dimensionless velocity (dimensionless)
*N*	Microorganism concentration (m^−3^)	*N*_1_	Navier slip parameter (dimensionless)
*Le*	Lewis number (dimensionless)	*n*	Number of factors (dimensionless)
*u*_*e*_	Velocity far from the surface (m/s)	*N*_*w*_	The concentration of microorganisms in close proximity to the surface (m^−3^)
k*	Mean absorption coefficient (m^−3^)		
*P*	Quantity of central points (dimensionless)	*N*_∞_	Concentration of microorganisms at a significant distance from the surface (m^−3^)
Wc	Microorganism maximum swimming speed (m/s)		
*Pe*	Peclet's number for bioconvection (dimensionless)	*Pr*	Prandtl number (dimensionless)
*q*_*m*_	Flux of mass across the wall (kg/s)	*T*	Temperature (K)
*q*_*n*_	Wall motile microorganism flux (kg/s)	*T*_*w*_	Temperature near the surface (K)
*q*_*w*_	Wall heat flux (*W*)	*T*_∞_	Temperature at a significant distance from the surface (K)
*u*, *v*	The velocity components in the x- and y-directions (m/s)	x, y	Cartesian coordinates (m)
*r*_0_	Intercept term (dimensionless)	*r*_*i*_	Linear term (dimensionless)
*r*_*ii*_	Quadratic term (dimensionless)	*r*_*ij*_	Bilinear term (dimensionless)
**Greek symbols**			
*α*	Nanoparticle thermal diffusivity (m^2^/s)	*β*	Parameter indicating the degree of thermal slip (dimensionless)
*γ*	The parameter that characterises the degree of velocity slip (dimensionless)	*r*	Dimensionless constant (dimensionless)
*η*	The variable of similarity (dimensionless)	*η*_∞_	Boundary layer thickness (m)
*θ*	The concept of temperature expressed in a non-dimensional form (dimensionless)	*μ*	Dynamic viscosity (Ns/m^2^)
*λ*	Shrinking parameter (dimensionless)	*υ*	Kinematic viscosity (m^2^/s)
*σ*_1_	Stream function (m^2^/s)	*σ*_1_	Electrical conductivity
*τ*_*w*_	Surface shear stress (N/m^2^)	*τ*	The ratio of the heat capacity of nanoparticles to that of the base fluid (dimensionless)
*ϕ*	The concentration of nanoparticles expressed in a dimensionless form (dimensionless)	*χ*	The density of microorganisms expressed in dimensionless units (dimensionless)
σ*	Stefan–Boltzmann constant		

## Conclusion

This study delves into the examination of a two-dimensional bio nanofluid flow model near a stagnation point on a contracting surface, considering various factors. The mathematical model takes into account the impact of velocity and thermal slippage at the contracting surface. Utilizing transformed ordinary differential equations, numerical simulations are conducted using the Homotopy Analysis Method (HAM). The investigation reveals that the presence of Couple stress and the heat source/sink parameter exerts a significant influence on the flow and heat transfer characteristics. Notably, an increase in the Couple stress and magneto-porous parameter leads to a corresponding rise in the skin friction coefficient. Additionally, it is worth highlighting that the rate of heat transfer decreases at higher values of the Prandtl number and the heat sink parameter, while it increases at higher values of the magneto-porous parameter, heat source, and coupling stress parameter. These findings carry important implications, particularly in applications where reducing skin friction and enhancing heat transfer are key objectives. The study also presents numerical data demonstrating the impact on the skin friction, Nusselt number, and Sherwood number.

## Data Availability

The data that support the findings of the study are available from the corresponding author upon reasonable request.
